# Microglia-Centered Combinatorial Strategies Against Glioblastoma

**DOI:** 10.3389/fimmu.2020.571951

**Published:** 2020-09-29

**Authors:** Tomás A. Martins, Philip Schmassmann, Tala Shekarian, Jean-Louis Boulay, Marie-Françoise Ritz, Steven Zanganeh, Johannes vom Berg, Gregor Hutter

**Affiliations:** ^1^Department of Biomedicine, University of Basel, Basel, Switzerland; ^2^Department of Neurosurgery, University Hospital Basel, Basel, Switzerland; ^3^Sloan Kettering Institute, Memorial Sloan Kettering Cancer Center, New York, NY, United States; ^4^Department of Chemical and Biomolecular Engineering, New York University, New York, NY, United States; ^5^Institute of Laboratory Animal Science, University of Zurich, Schlieren, Switzerland

**Keywords:** glioblastoma, immunotherapy, microglia modulation, glioma-associated microglia, glioma- associated macrophages, immune tumor microenvironment

## Abstract

Tumor-associated microglia (MG) and macrophages (MΦ) are important components of the glioblastoma (GBM) immune tumor microenvironment (iTME). From the recent advances in understanding how MG and GBM cells evolve and interact during tumorigenesis, we emphasize the cooperation of MG with other immune cell types of the GBM-iTME, mainly MΦ and T cells. We provide a comprehensive overview of current immunotherapeutic clinical trials and approaches for the treatment of GBM, which in general, underestimate the counteracting contribution of immunosuppressive MG as a main factor for treatment failure. Furthermore, we summarize new developments and strategies in MG reprogramming/re-education in the GBM context, with a focus on ways to boost MG-mediated tumor cell phagocytosis and associated experimental models and methods. This ultimately converges in our proposal of novel combinatorial regimens that locally modulate MG as a central paradigm, and therefore may lead to additional, long-lasting, and effective tumoricidal responses.

## Development and Classification of Glioblastoma

Glioblastoma (GBM) is the most aggressive and common primary brain tumor. Despite current treatment modalities, consisting of surgical resection followed by chemo-irradiation, the median overall survival of GBM patients remains only 15 months (1). These tumors arise from astrocytes or their precursors within the central nervous system (CNS) and are genetically and phenotypically heterogeneous (2). World Health Organization (WHO) grade IV glioma that arises *de novo* is designated primary GBM while that developing from the progression of previously diagnosed lower-grade glioma is named secondary GBM (3).

**Figure 1:**
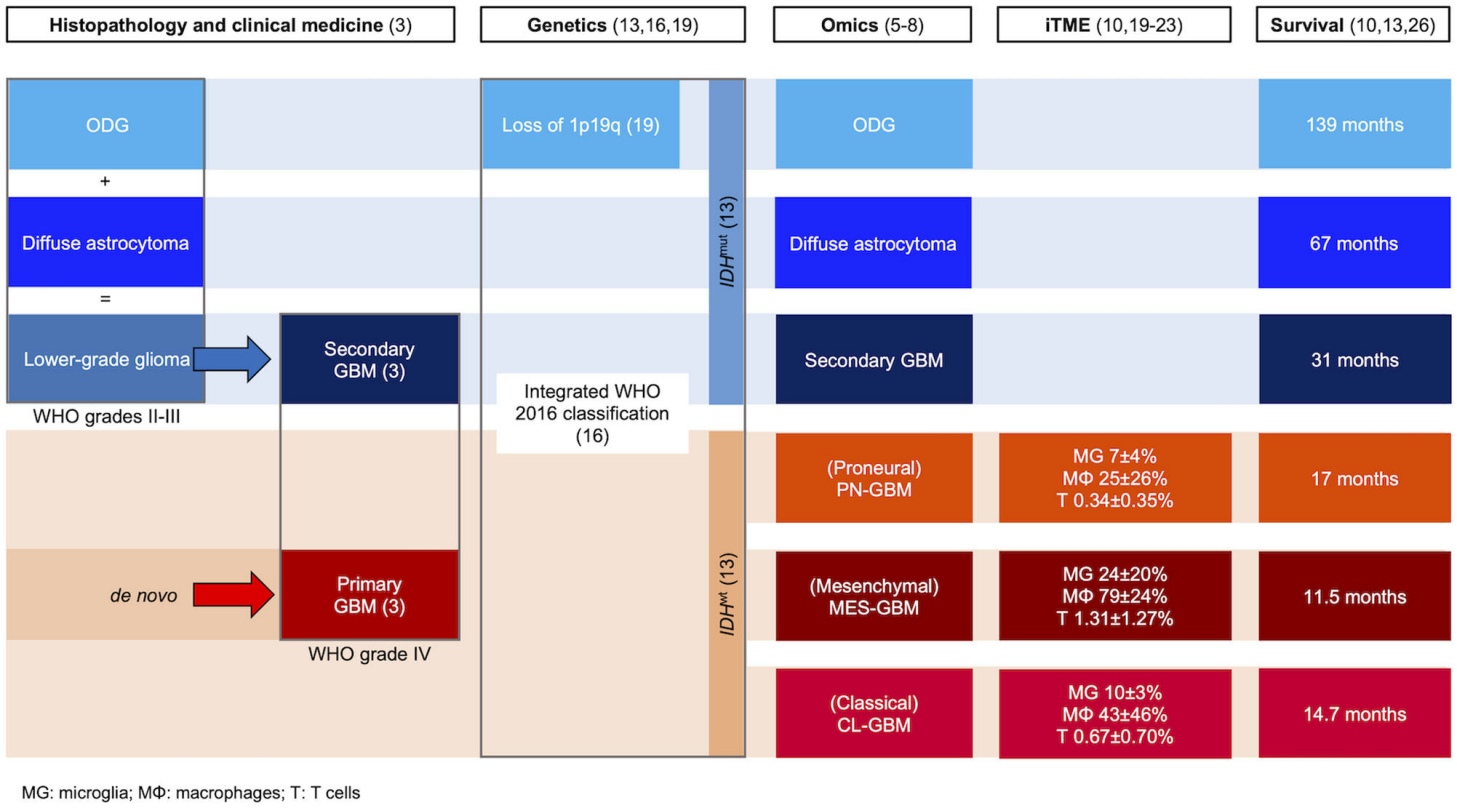
Classification of GBM based on histological, clinical, genetic, -omic, and immune features. *IDH*^mut^ and *IDH*^wt^ glioma subtypes are indicated in shades of blue and red, respectively. Arrows indicate tumor progression. The definition of iTME composition is described in the relevant publications. Median survival of ODG and diffuse astrocytoma is based on WHO grade II (3, 5–8, 10–19).

In the course of primary GBM development, chromosome 7 gain and chromosome 10 loss have led to the identification of platelet-derived growth factor subunit A (*PDGF*A) and phosphatase and tensin homolog (*PTEN*) as driver genes (4). Based on genomic, transcriptomic, and proteomic profiles, primary GBM has been further subclassified into classical (CL), proneural (PN), or mesenchymal (MES) subgroups (5–8). While CL-GBM shows frequent epidermal growth factor receptor (*EGFR*) amplification and cyclin-dependent kinase inhibitor 2A (*CDKN2A*) homozygous deletion, PN-GBM is associated with amplification of platelet-derived growth factor receptor alpha (*PDGFRA*) and tumor protein p53 (*TP53*) mutations. Finally, MES-GBM, is associated with additional loss of neurofibromin 1 (*NF1*) gene, and co-mutated *PTEN* and *TP53* tumor suppressor genes (4, 5). In sum, the genetic alterations that distinguish all 3 GBM subgroups commonly hit the same three major glioma signaling pathways: the RTK/RAS/PI3K (proliferation), TP53 (apoptosis) and RB (cell division) pathways (9). At the clinical level, MES-GBM shows the shortest median survival (11.5 months), compared to CL- and PN-GBM (14.7 and 17 months, respectively) (10) (Figure 1). Within these 3 GBM subgroups, limited therapeutic benefit has been observed (5, 6). Additionally, NFKB inhibitor alpha (*NFKBIA*) deletion confers radio-resistance in MES-GBM (20, 21).

Secondary GBM and its precursors harbor isocitrate dehydrogenase [NADP(+)] 1 (*IDH1*) and 2 (*IDH2*) mutations (collectively *IDH*^mut^), in addition to either *TP53* mutations in low-grade astrocytoma (LGA) and high-grade astrocytoma (HGA), or co-deletion of chromosome 1p/19q in oligodendroglioma (ODG) (11, 22). In contrast to *IDH*^wt^, glioma patients retrospectively identified as *IDH*^mut^ showed improved survival upon standard of care temozolomide (TMZ) treatment (23). Together with histopathology, *IDH* mutation and 1p/19q co-deletion statuses are now used resulting in the current integrated WHO classification (12). The classification of brain tumors into *IDH*^mut^ (HGA, LGA, and ODG) or *IDH* wild type (*IDH*^wt^; CL-, MES-, and PN-GBM) has been further supported by methylomics (8) (Figure 1).

## Implications of GBM Subtype on Immune Cell Infiltrates

GBMs frequently contain high proportions of non-neoplastic immune cells that collectively form the immune tumor microenvironment (iTME). The considerable number of immune cells within these tumors may account for the gene expression variability observed between GBM patient biopsies (24). Tumor-associated immune cells primarily enrolled for cytotoxicity against tumor cells, are typically hijacked by the tumor to promote its progression through mutual tumor-immune cell paracrine interactions and genetic reprogramming. Furthermore, the high content of macrophages (MΦ) and microglia (MG) and low frequency of lymphocytes in the GBM-iTME classify GBM as a lymphocyte-depleted tumor (25). Since studies describing the immune cell composition of glioma biopsies *in situ* have used distinct methodologies and calculation modes, interstudy comparison is not quantifiable. Nevertheless, the superimposition of those data shows consistent trends. First, *IDH*^wt^ primary GBM patients, having shorter overall survival relative to *IDH*^mut^ secondary GBM patients, show globally higher MG, MΦ, and T lymphocyte composition (13). Then, among *IDH*^mut^, from WHO grade II to IV secondary GBM, progressively reduced patient outcome correlates with increased MG, MΦ and T cell contents (14). Finally, primary GBM subgroups show differences in their immune composition, again linking tumor progression and reduced patient survival with higher proportions of immune cells (15–17). Importantly, *NF1* loss (MES subtype) resulted in increased glioma-associated microglia and macrophage (GAM) infiltration, which was even more pronounced in recurrent GBM (10) (Figure 1). Thus, there is convincing evidence of increased recruitment of tumor-associated immune cells during brain tumor development, suggesting an oncogenic contribution of the iTME. Hampering this paracrine symbiotic association may lead to greater control of tumor progression. Mechanistically, the accumulation of 2-hydroxyglutarate resulting from *IDH* mutations suppresses the accumulation and activity of infiltrating T cells by impairing the nuclear factor of activated T cells (NFAT) expression in a paracrine manner (26, 27). Further, NF-κB activation of GAMs mediates PN- to MES-GBM transition, while *NF1* inactivation, a hallmark of MES-GBM, results in higher numbers of infiltrating, anti-inflammatory, M2 GAMs and CD4^+^ memory T cells (10, 21).

In parallel to *IDH*^mut^ tumors and their hypermethylator phenotype, GBM can acquire a hypermutator phenotype resulting from TMZ-based chemotherapy (18, 28–31). Concurrently, the accumulation of neoantigens stimulates the recruitment of CD8^+^ T cells into the tumor (10). Thus, the occurrence of spontaneous or TMZ-induced tumor-specific neoantigens represents a potential modulator of iTME composition and T cell-mediated anticancer cytotoxicity in GBM. Altogether, the crosstalk between tumor and infiltrating immune cells suggests possible therapeutic interventions to redirect immune cells against neoplastic cells to further control glioma progression.

## Characterization of the GBM-iTME

The brain has historically been considered an immune-privileged organ (32, 33). This concept was long supported by three main observations: (a) the existence of a specialized vasculature in the brain, termed the blood-brain barrier (BBB) (34), (b) the lack of a conventional lymphatic system, (c) and a poorly characterized brain-specific immune cell population—MG. This classical dogma has been challenged by several studies that demonstrated that the CNS is in fact actively interacting with the immune system (35). Increasing evidence suggests that inflammation is the prime cause of many neurodegenerative diseases, and it is now generally accepted that the CNS undergoes constant intrinsic and peripheral immune surveillance (36–38). One such mechanism of immunosurveillance has been elucidated by the discovery of a CNS-specific lymphatic system. This study established that antigens and T cells can reach the cervical lymph nodes through cerebrospinal fluid-filled channels (39). In addition, antigens may also enter the cerebral arteries and cervical lymph nodes through the Virchow Robin perivascular spaces, and immunoglobulins are able to cross the BBB via carrier-mediated transport (40, 41). Taken together, these observations point toward the existence of important interactions between the CNS and the immune system, and underscore the role of the immune system in the induction and progression of brain cancers. Moreover, they emphasize the potential for immunotherapeutic approaches in the treatment of brain tumors.

The complex GBM-iTME is dominated by immunosuppressive cytokines such as prostaglandin E_2_ (PGE2), transforming growth factor beta 1 (TGFB1), and interleukin (IL)−6 and−10 (42, 43). Important “hubs of immunosuppression” such as high expression of STAT3 or FGL2 by GBM cells might directly act as paracrine mediators on the pleiotropic iTME, and could be universally targeted (44). In parallel, regulatory CD4^+^ T-helper cells (Tregs) are an important immune population in the GBM-iTME (45). Both natural Tregs (nTregs)—naturally occurring in the thymus—and induced Tregs (iTregs)—induced by activation with antigen or by antigen-presenting cells (APCs)—have been reported to contribute to GBM-mediated immunosuppression, with nTregs reportedly having a dominant role in the GBM-iTME (46). Cytotoxic CD8^+^ T cells are very rare, accounting for under 20% of all CD3^+^ lymphocytes, and appear loosely distributed in the GBM parenchyma (47). In an immunohistochemical (IHC) study of tissue microarray cores from 284 gliomas, the number of CD8^+^ tumor-infiltrating lymphocytes (TILs) correlated negatively with tumor grade whereas the number of CD4^+^ TILs displayed a positive correlation (48). Another recent study reported that GBM-TILs increased their expression of indoleamine 2,3-dioxygenase (IDO1), an enzyme that catalyzes tryptophan (TRP) degradation, resulting in the depletion of TRP in the local iTME and consequent inhibition of T cell responses (49). Moreover, another study demonstrated that GBM patients and GBM-grafted mice may harbor peripheral blood CD4^+^ T cell counts as low as acquired immune deficiency syndrome subjects and show T cell-deficient lymphoid organs. Concomitantly, large numbers of T cells were instead found sequestered in the bone marrow (BM), accompanied by tumor-imposed loss of sphingosine 1 phosphate receptor 1 (S1PR1) from the T cell surface (50).

Yet, perhaps the most notable aspect of the GBM-iTME is its population of tumor-associated MΦ and MG—collectively referred to as GAMs. These are the most abundant GBM-infiltrating immune cells and may contribute to up to half of the total tumor mass (51, 52). In addition to the recruitment of brain-resident MG to the tumor site, the high number of GAMs in glioma is a cumulative result of the influx of myeloid-derived MΦ into the brain as a consequence of tumor-induced neoangiogenesis and inflammatory stimuli. This inflammatory iTME acts in an immunosuppressive manner to promote tumor progression [e.g., via reprogramming of GAMs to anti-inflammatory states by paracrine tumor cell-GAM crosstalk (53–56)]. The contribution of GAMs to gliomagenesis continues to unveil the complex interactions of GBM cells with their microenvironment (53, 54, 57). Together, these data suggest that in addition to T cells, GAMs represent an attractive cell population with an intrinsic functional repertoire that may be reprogrammed to target tumor cells. In the Supplementary Information and Supplementary Table 1 of this review, we provide a comprehensive overview of the most recent clinical trials and their strategies in interfering with the innate and adaptive GBM-iTME.

## Distinction of BM-derived MΦ From Brain-Resident MG

MG are dynamic and specialized CNS-resident immune cells. Their name was first coined by Pío Del Río Hortega, then a student of Santiago Ramón y Cajal, and published in the Bulletin of the Spanish Society of Biology in 1919. MG are constantly monitoring the CNS and become activated in response to pathogens or CNS injury (58, 59).

Various experiments including parabiosis, adoptive transfer and fate mapping studies conducted in mouse models have elucidated our understanding of MG and their distinction from peripheral, BM-derived MΦ (51, 60–65). MG and MΦ are thus distinct and ontogenically different cell populations (54).

Despite the separate origins of MG and MΦ, GAM accumulation within and around GBM has raised interest in dissecting the roles of these cells in tumor progression. Many common chemoattractant factors have been identified for MG and MΦ (57). In the healthy brain, the CX3C motif chemokine receptor 1 (CX3CR1) is mostly expressed by MG and has been established as a reliable marker for MG imaging (57). Notably, a polymorphism in the *CX3CR1* gene has been associated with reduced tumor infiltration by MG which led to increased survival of GBM patients (66). Others reported conflicting findings regarding the importance of CX3CR1 and its ligand—CX3C motif chemokine ligand 1 (CX3CL1)—in tumor-directed MG migration (67, 68). However, infiltrating monocytes, differentiating into MΦ express it as well, implying that CX3CR1 does not represent a MG-specific marker, especially in the context of glioma (67). Notably, a recent study identified perivascular, meningeal, and choroid plexus MΦ as non-parenchymal brain MΦ that mediate immune responses at the brain boundaries and, like MG, express CX3CR1 in the healthy brain (69). One of the first chemoattractant factors identified is CC motif chemokine ligand 2 (CCL2) or MCP1. Ectopic expression of CCL2 in rat glioma cells showed increased tumor growth, with massive infiltration of MG/MΦ, resulting in reduced survival (70). Interestingly, it has been recently described that in mice, MG, in contrast to MΦ, do not express the CCL2 receptor, CC motif chemokine receptor 2 (CCR2), providing a novel model to investigate monocyte subset trafficking within the GBM-iTME (71). In fact, Hutter and colleagues used a *Ccr2* knockout mouse model which limits MΦ infiltration into the tumor site, enabling the specific study of MG within the GBM iTME (72). Colony stimulating factor 1 (CSF1) or M-CSF is another potent GAM-recruiting cytokine. Blocking its receptor, colony stimulating factor 1 receptor (CSF1R) reduced GAM density and attenuated GBM invasion *in vivo* (73, 74). Similar results were reported by a knockdown of its close relative, CSF2, which resulted in reduced MG-dependent invasion in organotypic brain slices as well as diminished growth of intracranial gliomas accompanied by extended survival in animal models (75).

Approaches to distinguish these cell populations have traditionally relied on the expression of the hematopoietic marker CD45, with yolk sac-derived MG being CD45^low^ and infiltrating MΦ of hematopoietic origin CD45^high^ (76). This paradigm has been recently challenged by a study using irradiated chimeras with head protection which impeded the massive unspecific influx of monocytes due to a disrupted BBB. The authors showed that MG are able to upregulate CD45 and represent an inherent part of the CD45^high^ population in the tumor context (77).

Therefore, better targets are needed to accurately distinguish resident MG from infiltrating inflammatory monocytes and non-parenchymal brain MΦ to better understand their contribution in glioma formation, maintenance, and progression.

In a traumatic brain injury model, *in vivo* time-lapse 2-photon imaging of MG revealed their rapid and targeted migration and process extension to the site of injury, establishing a barrier between the healthy and injured tissue. This rapid chemotactic response is mediated by the release of nucleotides following CNS injuries (59). MG express several G protein-coupled receptors, including the G protein-coupled purinergic receptor P2Y 12 (P2RY12), a putative primary site where nucleotides act to induce MG chemotaxis. P2RY12 is also expressed on platelets and required for normal platelet aggregation and blood coagulation (78). In the brain parenchyma, its expression is well-limited to MG, making it a very useful marker in MG identification (79). Another useful marker to distinguish MG from infiltrating MΦ is integrin subunit alpha 4 (ITGA4) or CD49D, which was specifically repressed in the MG of different mouse models of glioma. Its translational relevance has also been shown in human GBM biopsies (53).

Recent advances in RNA sequencing and other cell profiling technologies have enabled the discovery of cell-type-specific signature genes. Among these, a transmembrane protein of unknown function—transmembrane protein 119 (TMEM119)—is exclusively expressed by MG in the human and mouse brain (80). Hence, TMEM119-specific antibodies are now widely used in IHC and flow cytometric (FC) applications. The ongoing large-scale transcriptional profiling of MG further identified novel cell lineage-specific genes like hexosaminidase subunit beta (*HEXB*), which is highly expressed in MG and encodes a subunit of the lysosomal enzyme hexosaminidase, that catalyzes the degradation of gangliosides (81). These novel instruments for cell-specific tracking and genetic modulation will enhance the specificity and sophistication of MG studies as well as our understanding of MG functions in the context of glioma.

## MG Activation and Immune Cell Interactions in the GBM-iTME

MG accumulated within GBM typically undergo a morphological transformation from a ramified, resting phenotype, to an amoeboid, activated state (51). For MΦ, different types of activation have been defined following *in vitro* stimulation. The pro-inflammatory M1 phenotype is typically acquired after stimulation with IFNγ, alone or in concert with microbial cues such as LPS. Whereas, anti-inflammatory molecules, such as IL-4, -10, and -13, are inhibitors of MΦ activation and induce the alternative M2 phenotype (82, 83). These polarized MΦ subpopulations differ in terms of receptor expression, effector function, and cytokine and chemokine production (83). Given that these definitions of the different activation states are based on *in vitro* conditions, and the M1 and M2 phenotypes represent the extremes of a broader spectrum of functional states, they are only to some extent translatable to the *in vivo* settings. In the era of single cell sequencing and mass cytometry, and much more detailed functional state analysis, this polarization classification may soon become obsolete in the MG field. Nevertheless, several studies have analyzed the expression of M1 and M2 markers among GBM-associated GAMs and concluded that, similarly to other solid tumor types, they predominantly exhibit an anti-inflammatory M2 polarization and reduced phagocytic activity (54, 84–87). It is believed that glioma-derived molecules such as CSF1 induce the shift of MG and MΦ toward the M2 phenotype and thus create a favorable microenvironment for GBM growth (86). In addition, GAM expression of CD163 and macrophage scavenger receptor 1 (MSR1) or CD204, both of which are considered M2 MΦ markers, was significantly higher in grade IV GBM when compared to low-grade glioma (LGG), indicating that polarization of GBM-associated MG and MΦ toward the M2 phenotype correlates with a more malignant histological grade (55). Accordingly, others identified the expression of CD74, an M1 polarization marker, by human GAMs to be positively correlated with the overall survival of GBM patients (88). However, useful they may have been in establishing and dissecting the functions of MG, the traditional M1 and M2 phenotypes, and the resulting classification of MG responses into a binary system of pro- or anti-inflammatory has so far produced an oversimplified insight to their complex roles in the context of brain diseases (89, 90).

Studies of human and murine neurodegenerative diseases, as well as brain tumors, have identified genes and their encoded proteins previously known to be expressed in the DC compartment of the peripheral immune system. Moreover, transcriptomics data from diverse neurodegenerative disease studies show MG upregulation of genes involved in APC-T cell interactions (91). Interestingly, similar trends have been found in MG isolated from GL261 syngeneic GBM mouse models as well as in tumor biopsies of GBM patients. This upregulated gene set included human and mouse homologs of immunosuppressive modulators (C type lectin domain containing 7A, *CLEC7A*; glycoprotein nmb, *GPNMB*; leukocyte immunoglobulin like receptor B4, *LILRB4*; and *PDCD1*) as well as stimulators (integrin subunit alpha X, *ITGAX* or *CD11C*; and secreted phosphoprotein 1, *SPP1*) of the adaptive immune system. Collectively, these studies show that MG derived from tumor and neurodegenerative states both contribute to immunosuppression and altered T cell responses in the brain (92–94).

In fact, a recent study showed that in the context of Alzheimer's disease (AD), chronically activated MG limit CD3^+^/CD8^+^ T cell recruitment to the brain (95). Another study with GL261 murine glioma models demonstrated that MG are functional APCs and are required for complete antigen-specific CD8^+^ T cell responses in an MHC class I-dependent manner (96). Given the parallels between the inflammatory states resulting from brain tumors and neurodegenerative diseases, a better understanding of the link between innate and adaptive immune responses in the brain in combination with an improved characterization of MG heterogeneity, remain future directions for targeted immunotherapies against GBM.

Recently, combined high-throughput technologies of regionally annotated MG cells and intratumoral MG have mapped specific functional differences of MG in healthy vs. GBM-burdened brains. In non-neoplastic brains, nine clusters of heterogeneous MG functional states were identified whereas in GBM-associated MG, single-cell RNA sequencing (scRNA-seq) revealed even more heterogeneity–15 clusters—with upregulation of pro-inflammatory and metabolic genes, including *SPP1*, and several type I interferon genes, including apolipoprotein E (*APOE*) and *CD163*. By concurrent mass cytometry, the upregulation of HLA-DR, triggering receptor expressed on myeloid cells 2 (TREM2), APOE, adhesion G protein-coupled receptor G1 (ADGRG1) or GPR56, solute carrier family 2 member 5 (SLC2A5) or GLUT5, and Fc fragment of IgG receptor Ia (FCGR1A) or CD64 was confirmed in GBM-associated MG vs. normal control MG (97). This underscores the diversity and plasticity of MG in the healthy brain and the GBM-iTME, and reiterates the difficulty in targeting these cells for treatment.

## MG in GBM Progression

Early co-culture studies noted that the motility of murine glioma cells was increased in the presence of MG, and that this glioma-promoting effect could be further enhanced by MG-activating substances like CSF2 (98). GBM cells are known to constitutively release CSF1 and CSF2, which act as chemoattractants for MG and convert GAMs to protumoral phenotypes (74). Consistent with the tumor-promoting effect of CSF1, blockade of CSF1R led to decreased expression of M2 markers in GAMs, resulting in regression of established tumors and increased survival in a mouse GBM model (74). To summarize, once MG and MΦ are recruited to the tumor site and re-educated to a protumorigenic phenotype, mutual paracrine signaling between GAM and GBM cells is established whereby glioma growth and invasion are promoted. Similar effects on glioma cells could be shown by using GAM-conditioned media instead of co-cultures (98). Many of the soluble factors involved in GAM-glioma crosstalk have been identified, such as epidermal growth factor (EGF), which is released by MG and stimulates GBM cell migration and invasion via the commonly upregulated epidermal growth factor receptor (EGFR) on glioma cells (73). Other factors include anti-inflammatory TGFB1 and IL-10, pro-inflammatory molecules like TNF, IL-1B, and IL-6, as well as pro-angiogenic factors like vascular endothelial growth factor A (VEGFA). TGFB1 promotes the migration of glioma cells via processes that likely involve the upregulation of integrin expression and function (99). Furthermore, TGFB1 induces the release of matrix metallopeptidase 2 (MMP2) in its inactive form—pro-MMP2—which becomes activated upon cleavage by the membrane-bound matrix metallopeptidase 14 (MMP14) (99, 100). GBM-associated MG upregulate MMP14 and thereby facilitate the invasion of glioma cells into the brain parenchyma by metalloproteinase-mediated degradation of the extracellular matrix (100). A recent study by Walentynowicz et al. sought to assess the role of human GBM conditioned media on human MG cell lines on the MG transcriptome. *TGM2* and *GPNMB* were identified across various datasets, but their relevance is awaiting further experimental validation (101).

Along with this paracrine glioma-promoting effect, GAMs also enable GBM engraftment and invasion by failing to efficiently eliminate cancer cells by phagocytosis. Their role as phagocytic innate immune cells is perturbed by glioma cells rendering MG and MΦ to an anti-inflammatory, antiphagocytic M2 phenotype (102). Moreover, upregulation of the so-called “don't eat me” signals on the surface of glioma cells and masking of antigenic sites by overexpressing sialic acid-rich glycoproteins are both effective strategies to inhibit phagocytosis and evade innate immune surveillance (103–105).

## Modeling MG-GBM Interactions

The generation of a mouse strain in which the *Cx3cr1* locus was replaced by a green fluorescent protein (GFP) reporter gene (*Cx3cr1*^+/*GFP*^) allowed for the first time the direct study of MG *in vivo* using 2-photon-microscopy (106, 107). This mouse line strongly labels MG and is the best-studied model in MG research (106, 108). To further exploit the *Cx3cr1* promoter activity, the *Cx3cr1* gene was replaced with sequences encoding either Cre recombinase (*Cx3cr1*^*Cre*^) or a Cre recombinase fused to a mutant estrogen ligand-binding domain that requires the presence of the estrogen antagonist tamoxifen for activity (*Cx3cr1*^*CreERT*2^) (109). These mouse lines enabled a conditional, MG-specific constitutive or inducible gene knockout, which advanced the specificity of MG research significantly (Table 1).

**Table 1:** Current MG mouse models.

**Target gene**	**Modifications**	**References**
*Cx3cr1*	*Cx3cr1^+/*GFP*^*	(106)
	*Cx3cr1^*GFP*/*GFP*^*	
	*Cx3cr1^*Cre*^*	(109)
	*Cx3cr1^*CreERT*2^*	
*P2ry12*	*P2ry12^−/−^ Cx3cr1^+/*GFP*^*	(79)
*Sall1*	*Sall1^*GFP*^*	(114)
	*Sall1^*CreERT*2^*	(115)
*Tmem119*	*Tmem119^*EGFP*^*	(116)
	*Tmem119^*CreERT*2^*	

Even though CX3CR1 is highly expressed on MG, it is expressed as well on MΦ, monocytes, and DCs (106). P2RY12, on the other hand, was initially investigated for its function as a regulator of platelet adhesion and activation. P2RY12-deficient mice were therefore primarily used to study platelet physiology and blood coagulation (110, 111). Eventually, P2RY12 was identified as a MG-specific marker in the brain parenchyma and *P2ry12*^−/−^ MG reporter mice were generated, allowing the study of P2RY12-mediated MG chemotaxis to the site of BBB injuries (79, 112) (Table 1).

Gene expression profiling not only identified MG specific surface proteins but also MG signature genes such as spalt like transcription factor 1 (*Sall1*), which encodes a transcriptional regulator (113). Accordingly, the introduction of *Sall1*^*GFP*^ and *Sall1*^*CreERT*2^ knock-in mouse lines represent more distinct models for MG tracking and genetic modulation *in vivo* (114, 115). The ongoing efforts, mainly based on large-scale transcriptional analysis of MG cells, will keep providing novel targets for even more specific *in vivo* imaging and modulation. Very recently demonstrated by the discovery of TMEM119 which was shortly followed by the introduction of a knock-in *Tmem119*^*EGFP*^ reporter mouse line and *Tmem119*^*CreERT*2^ mice (80, 116) (Table 1).

With the increased interest in MΦ-focused immuno-oncology, assays that robustly and reproducibly determine the prophagocytic effect of a therapeutic agent of interest, are constantly evolving as well. While the first reports of the beneficial effect of CD47 disruption in leukemia cells, were mainly based on classical fluorescence microscopy, calculating the phagocytic index by dividing ingested cells by the total number of MΦ, they were soon replaced by FC-based approaches to better identify also smaller effect sizes in other tumor models (103, 117–121). In these experiments, phagocytes were identified by specific markers and co-incubated with cell-dye labeled tumor cells. MΦ that had successfully phagocytosed tumor cells were also positive for the tumor cell stain. However, this method lacks the optical confirmation that the tumor cell has been really engulfed by the phagocytic cell, which is why many studies still included a microscopic assessment or use more elaborately time-lapse live-cell microscopy which offers not only spatial but also temporal information (122). Technological advances enable the better identification of phagocytic events as well, as seen with the introduction of imaging FC, which combines the high throughput analysis of FC with the detailed morphometric information of fluorescence microscopy (123). Besides these *in vitro* phagocytosis assays, many efforts are undertaken to make the complex interplay between tumor cells and phagocytes visible. In many studies, after a specific treatment *in vivo*, the tumor mass is resected and dissociated and within the single-cell suspension, phagocytosis is measured as the ratio of the double-positive MΦ population by FC (118, 120, 122). This approach compared to *in vitro* models allows for a better understanding of the complex interface between innate and adaptive immune systems as they orchestrate the antitumor immune response together (124). More sophisticated and direct approaches employ specific reporter mice that enable *in vivo* imaging using 2-photon microscopy. As shown in their recent publication, Hutter et al. were able to demonstrate real-time phagocytosis of living glioma cells by MG and MΦ upon CD47 disruption using *Ccr2*^+/*RFP*^ *Cx3cr1*^+/*GFP*^ reporter mice, allowing the direct study of these cells in the TME (72). As new targets in innate immunotherapy are emerging, sophisticated methods will be needed to validate their prophagocytic capacity and clinical potential in cancer therapy, such as 3D cultures and tissue culture bioreactors for improved *ex vivo* tissue preservation (125). Another promising technology to study cell interactions, tissue composition, and spatial distribution of the iTME is high-dimensional multiplexing—CO-Detection by indEXing (CODEX)—that allows *in situ* tissue cytometry with the detection of over 50 parameters (126).

## MG Targeting and Modulation

As the largest immune cell population and one that positively correlates with glioma malignancy, invasiveness, and grade, MG represent the primordial target for modulation and antitumor immunotherapy. In this context, most strategies so far aimed at impairing GAM recruitment to the tumor site, thereby preventing their glioma-promoting effects. This included the previously mentioned blockade of CSF1R, disruption of periostin (POSTN), which is secreted by GSCs, and recruits GAMs through integrin α_v_β_3_ signaling, or inhibition of the CXC motif chemokine receptor 4 (CXCR4) chemotactic pathway (53, 74). The latter has been mainly implicated in MΦ mobilization through increased CXC motif chemokine ligand 12 (CXCL12) expression after radiation therapy (127). In combination with radiotherapy, a small molecule inhibitor of CXCL12/CXCR4 interactions prevented GAM infiltration and tumor recurrence (128). Another approach aimed at reversing the MG tumor-promoting effects and re-educating them to an antitumor phenotype. One report showed that activated NK cells combined with an antibody against chondroitin sulfate proteoglycan 4 (CSPG4) on GBM cells, were able to reverse the GAM phenotype (129). Osteopontin (OPN/encoded by *SPP1*) is another promising candidate protein secreted by GBM cells, which has prognostic implications and drives the protumorigenic reprogramming of MG, which can be therapeutically targeted (130, 131).

Recently, the focus has shifted toward the phagocytic role of MG as part of innate immune surveillance, most often targeted through the CD47/signal regulatory protein alpha (SIRPA) and the sialic acid/sialic acid binding immunoglobulin like lectin (SIGLEC) phagocytosis axes. CD47 is a widely expressed transmembrane protein with numerous functions, among which the inhibition of phagocytosis (132). Upon binding and activating its receptor SIRPA on the surface of mononuclear cells, CD47 inhibits the phagocytic activity of MΦ and MG (133). This antiphagocytic signal is transmitted via phosphorylation of the immunoreceptor tyrosine-based inhibitory motif (ITIM) on the cytoplasmic tail of SIRPA. Subsequent binding and activation of the protein-tyrosine phosphatase non-receptor type 6 (PTPN6) and 11 (PTPN11) blocks phagocytosis, putatively by preventing the accumulation of myosin-IIA at the phagocytic synapse (134). However, CD47 expression is best characterized for its role in hematopoietic cell homeostasis, particularly in red blood cells and platelets, where it is required to prevent their elimination by splenic MΦ. CD47 is thus considered a marker of self (133). In pathological processes, inflammation-mobilized hematopoietic stem cells protect themselves from phagocytosis by upregulating CD47 on their surface (117). This CD47 overexpression is co-opted by tumor cells and represents a common feature of hematologic and solid tumors, allowing them to evade innate immune surveillance (103, 117–119).

As a major “don't eat me” signal, CD47 is highly upregulated on the surface of nearly all human tumor cell types, including GBM cells. Transcriptional analysis of glioma patients revealed that high *CD47* mRNA expression levels were associated with decreased progression-free and overall survival, suggesting that *CD47* expression levels may serve as a clinically relevant prognostic factor (103). Willingham et al. were the first to describe the GAM re-educating effect of CD47 blockade in models of GBM. Using targeted monoclonal antibodies against CD47 enabled MΦ-dependent phagocytosis of patient-derived GBM neurospheres *in vitro*. Furthermore, the administration of anti-CD47 antibodies inhibited tumor growth and increased the survival of orthotopic immunodeficient mice transplanted with patient derived GBM cells, providing the first preclinical validation of CD47 as a therapeutic target in GBM (103). Additional studies showed that anti-CD47 treatment repolarized GAMs *in vivo* to an M1 phenotype and that both M1- and M2-polarized MΦ alike displayed a higher GBM cell phagocytosis rate under anti-CD47 treatment (120). The therapeutic safety and efficacy of anti-CD47 treatment was also demonstrated in mouse models of murine high-grade glioma as well as five aggressive and etiologically distinct human pediatric brain tumors (medulloblastoma, atypical teratoid/rhabdoid tumor, primitive neuroectodermal tumor, pediatric GBM, and diffuse intrinsic pontine glioma) (121).

More recently, Hutter et al. dissected the response of MG and infiltrating peripheral MΦ upon anti-CD47 treatment in GBM. Using a mouse model with genetically color-coded MΦ (*Ccr2*^*RFP*^) and MG (*Cx3cr1*^*GFP*^), they showed that even in mice lacking Ccr2-mediated MΦ recruitment to the brain (*Ccr2*^*RFP*/*RFP*^ *Cx3cr1*^*GFP*/+^), MG-mediated GBM phagocytosis was sufficient to reduce tumor burden and prolong survival under anti-CD47 treatment. This observation led to the identification of MG as effector cells of GBM cell phagocytosis in response to CD47 blockade (72).

Comparable to CD47 overexpression, the aberrant glycosylation of cancer cells represents a common feature of malignant transformation (135, 136). These glycoproteins and glycolipids are often terminated by negatively charged sialic acids. Sialic acids are derivatives of neuraminic acid, and the predominant sialic acid found in mammalian cells bears at its amino site an acetyl group, therefore termed *N*-acetyl-neuraminic acid. The addition of sialic acids is mediated by sialyltransferases, a family of glycosyltransferases (137). Hypersialylation, meaning the upregulation of sialic acid-containing glycans (sialoglycan) on the cell surface through altered sialyltransferase expression and the increased introduction of non-human sialic acids like *N*-glycolyl-neuraminic acid (xenosialylation) are, together with the altered glycosylation itself, key changes of malignant tissue and important for cancer progression (138, 139).

Sialic acids can modulate the iTME through SIGLEC engagement. To date, 14 human and nine mouse SIGLECs have been identified, differing in their sialic acid ligand specificity and intracellular signaling cascades. SIGLECs are expressed on most cells of the immune system and can transmit immunosuppressive signals upon binding to sialic acids. Similar to the inhibitory SIRPA receptor—inhibitory SIGLEC receptors contain ITIMs in their intracellular domain that signal negatively via the recruitment of PTPN6 and 11 (140). The physiological role of SIGLECs to recognize sialic acids as self-associated patterns and therefore counter-regulate overshooting immune reactions and limit tissue damage during inflammation can be exploited by cancer cells (141). Hypersialylation of tumor cells can thus contribute to tumor immune evasion (104).

Initially, immunoinhibitory SIGLECs in brain pathologies were primarily associated with CD33 or SIGLEC3 as a genetic risk factor for AD (142–144). Subsequent functional studies showed that CD33 inhibits MG uptake of amyloid-β plaques in diseased brains (145). More recently, CD22 or SIGLEC2 was also identified as a negative regulator of phagocytosis that is upregulated on aged MG. Inhibition of CD22 promoted the clearance of myelin debris, amyloid-β oligomers, and α-synuclein fibrils in an AD model (146). Other studies identified important roles of SIGLECs in neuro-inflammatory diseases, where immunoinhibitory SIGLECs convey neuroprotective functions by alleviating especially MG neurotoxicity (147, 148).

With the paradigm shift in cancer therapy that came with the discovery of immune checkpoint inhibitors, the sialoglycan-SIGLEC pathway attracted recently a great deal of attention as a novel target for cancer immunotherapy. This holds especially true in brain malignancies, since phase II and III clinical trials of classical immunotherapeutic agents like PDCD1 and CD274 inhibitors showed no significant improvement in the median overall survival of GBM patients (149). Correlative single-cell transcriptomic analysis, including The Cancer Genome Atlas (TCGA) data, showed that most members of the SIGLEC family are differentially expressed in glioma. Interestingly, several SIGLEC receptors are predominantly expressed on MΦ and GAMs with higher expression levels observed in high-grade gliomas (150).

In a more translational approach, others investigated the role of immunomodulatory SIGLECs in the treatment with glucocorticosteroids, including dexamethasone, which is frequently used to control tumor-induced edema in brain tumor patients. They found alterations in tumor cell surface sialylation and SIGLEC recognition in response to dexamethasone treatment (151). Specifically, MG showed an upregulation of SIGLEC receptors together with induction of an anti-inflammatory cytokine profile, indicating a crucial role of SIGLECs in dampening the dexamethasone-induced antitumor immunity (152). The first experimental evidence that linked SIGLECs with whole tumor cell phagocytosis in glioma dates back to 2013, when Siglec H, a MG-specific marker, was suggested to be a phagocytic receptor for glioma cells (153–155). Novel insights into the sialic acid-SIGLEC antiphagocytic axis have recently emerged. In particular, SIGLEC10 was identified as the receptor of CD24, an additional “don't eat me” signal. Tumor-expressed CD24 promoted innate immune evasion through its interaction with GAM-expressed SIGLEC10 (122). Another study focused on SIGLEC15 as an immune suppressor and potential target for cancer immunotherapy. Using a genetic mouse model and intracranial injection of murine glioma cells, the authors found significantly slower tumor growth associated with more MΦ and CD8^+^ T cells in the TME upon genetic ablation of SIGLEC15. Together with *ex vivo* restimulation assays, their data support a role for SIGLEC15 in MΦ-mediated suppression of tumor immunity (156). The mounting evidence of SIGLEC engagement by cancer cells to evade the antitumor immune response, especially innate immune response, make sialic acid-SIGLEC interactions very attractive candidates for potentiating antitumor immunity in GBM.

## Discussion: Emerging Local and Combinatorial Approaches for the Treatment of GBM Place MG at the Center Stage

Despite advances in surgical techniques, radiation therapy, and chemotherapy, effective treatment of GBM remains an unresolved challenge. Today's unspecific approach of alkylating chemotherapy and radiation therapy causes major toxicities and debilitating side effects. Better ways to control this devastating disease are urgently needed.

We previously showed that modulation of MG within GBM (e.g., by CD47-SIRPA disruption), can control GBM progression by rendering MG tumor-phagocytic. Although disrupting CD47-SIRPA modulates MΦ and MG anti-GBM activity and reprograms the immunosuppressive iTME, GBM represents a heterogeneous tumor entity with a multitude of deregulated cancer pathways. Therefore, a subset of tumor cells will evade the MG-mediated antitumor response and develop resistance. We are thus convinced that reprogramming of MG within the tumor will not suffice by itself to halt GBM entirely, especially in view of emerging insights into MG heterogeneity. On the other hand, pure tumor-targeting approaches, vaccinations with tumor antigens, monoantigenic CAR T cells, or intratumoral cytokine deliveries are all prone to failure because of the overwhelming immunosuppressive contribution of the iTME, and specifically tumor-educated MG. Therefore, more sophisticated combinatorial approaches that target MG, adaptive immunity, and tumor cells at once are mandated.

**Figure 2:**
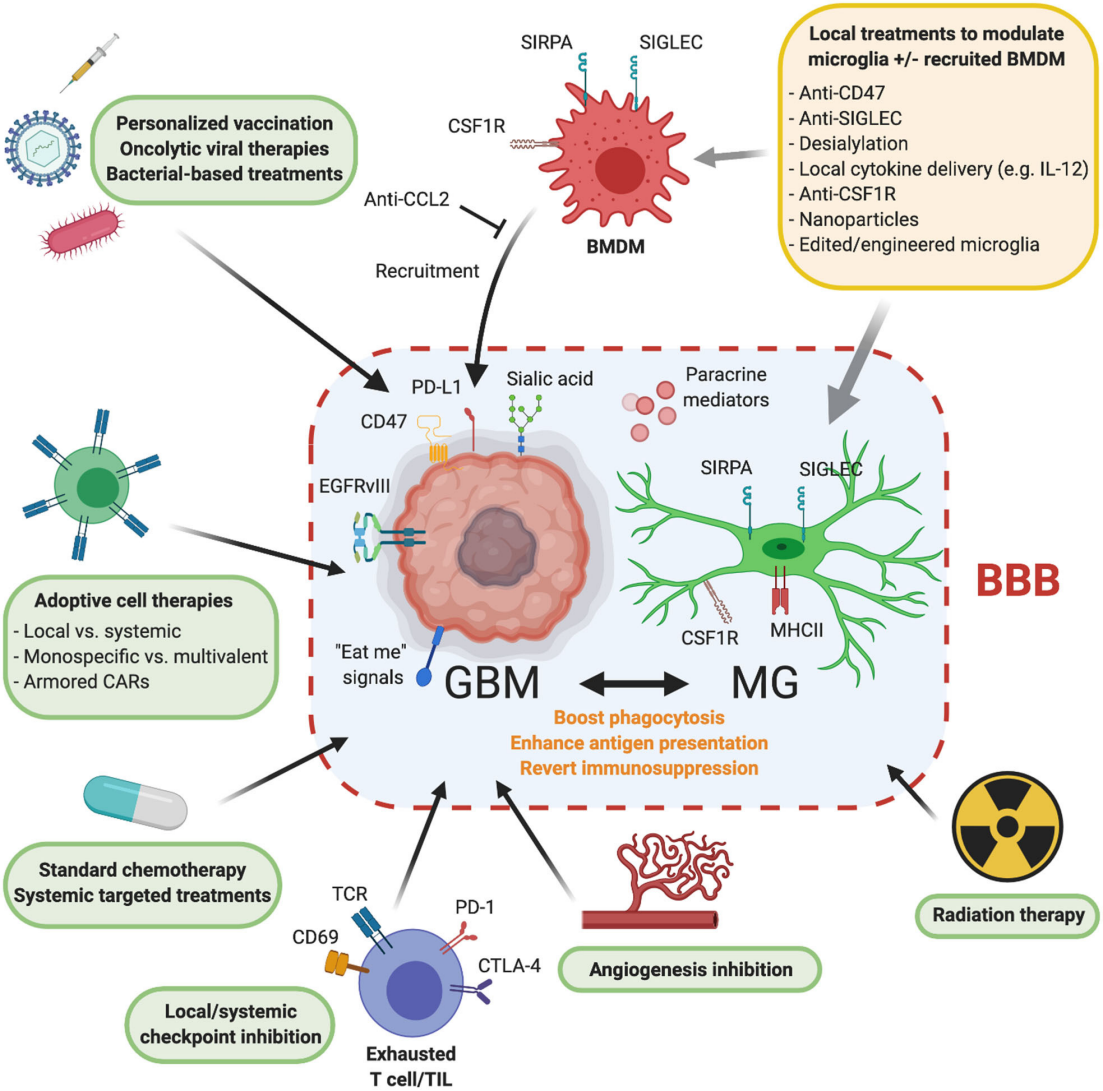
Combinatorial approaches of local MG modulation and other treatment modalities against GBM. Means of local MG modulation (upper right): MG can be targeted locally beyond the BBB by various approaches to influence MG phagocytosis, enhance antigen presentation, and revert generalized immunosuppression. Prominent potential means to locally modulate MG include anti-CD47 or anti-Siglec treatments, intratumoral desialylation, application of pro-inflammatory cytokines, “reprogramming” by blocking CSF1 signaling, or addition of immunomodulatory nanoparticles. Some of these modalities would also interfere with infiltrating BMDMs (e.g., anti-CCL2 blockade). Overall, this would enable other tumor specific or immunotherapeutic regimens to exert their antitumorigenic activity. Combinatorial approaches with MG modulation: *Personalized vaccination, oncolytic viral therapy, or bacterial based approaches*: the effect of tumor-specific vaccinations, oncolytic viruses, or tumor-targeting bacteria might be significantly enhanced when tumor-associated MG is reverted to a less immunosuppressive phenotype. *MG modulation* + *tumor-targeting CAR T cell therapy:* combining MG modulation with tumor antigen-specific CAR T cell therapy poses another way to circumvent current obstacles in GBM therapy. Local application of MG modulation and tumor-specific CAR T cells might result in better GBM control. Novel CAR T products could combine MG modulation (e.g., by reprogramming MG and targeting the tumor at once). *MG modulation* + *chemotherapy or targeted treatments:* tumor cells respond to established chemotherapy with increased expression of “eat-me” signals. Combinatorial strategies of already established chemotherapies with inducers of MG phagocytosis could improve treatment responses. *MG modulation* + *T cell checkpoint therapy:* this dual strategy of targeting the major players of the GBM-iTME—MG—and facilitating an intratumoral T cell response in addition to a putative MG-mediated T cell response might boost tumor regression. Further, the thorough analysis of tumor-phagocytosing MG vs. non-phagocytosing MG, their MHC molecules and linked, presented antigens, could lead to the discovery of novel tumor antigens and result in potential vaccination candidates. *MG modulation* + *angiogenesis inhibition:* anti-VEGFR treatment serves as a salvage therapy in recurrent GBM. Additional MG activation might prevent development of early resistance. *MG modulation* + *radiation therapy:* in line with chemotherapy, radiation therapy enhances immune responses and upregulates “eat-me” signals on tumor cells. Additional MG modulation could increase efficacy and long term treatment responses. Created with Biorender.com.

We believe that MG are at the centerstage for modulation in the iTME, since this will also influence the antitumoral capacity of other components of the iTME such as TILs [e.g., via enhanced antigen presentation (157)]. The capacity to of MG to present antigens (e.g., after tumor cell phagocytosis) needs to be evaluated further and with novel techniques in various experimental contexts since this might offer key insights into potential combinatorial strategies with vaccination studies or T cell checkpoint inhibitor treatments. How MG modulation and reprogramming is best achieved, and which—often redundant—immune evasion mechanisms should be targeted to achieve a durable induction of antitumoral activity is largely unknown. On top, the additional MΦ modulation and recruitment effects caused by the treatments should be considered, since additional recruitment of BM-derived MΦ might cause increased unwanted side effects such as enhanced edema. Prophagocytic pathways beyond CD47-SIRPA with higher MG specificity might be particularly attractive to tailor the MG response. However, an overshooting MG induction might as well-lead to deleterious effects in the brain (e.g., via hyperphagocytosis), and treatment effects, timing, and delivery need to be carefully validated in future clinical trials. Since most systemic treatments in brain tumors do not effectively reach the tumor because of the BBB, local/continuous application of these treatment regimens might be most effective, and application of these treatments in the early phase of the disease would be preferable over the post-treatment recurrent situation, where the iTME and tumor resistance mechanisms are even more deranged. Besides that, it remains to be studied, whether these treatments should be applied before or after tumor resection, and whether targeting of the peripheral invasion zone of the tumor, where presumably a lot of iTME reprogramming happens, might be advantageous. A multitude of strategies for MG modulation may unleash the inherent antitumoral armamentarium of MG and have translational potential; future translational research and clinical trials should pave the way on how to optimally design these approaches against GBM. In Figure 2, we summarize promising combinatorial treatment strategies to overcome these challenges.

## Author Contributions

GH and TS: conception or design of the work. TAM, PS, and TS: literature collection. TAM, PS, TS, J-LB, M-FR, JvB, SZ, and GH: drafting the article. GH: critical revision of the article. All authors final approval of the version to be published.

## Conflict of Interest

The authors declare that the research was conducted in the absence of any commercial or financial relationships that could be construed as a potential conflict of interest.
