# Supplementary material for: Microglia-Centered Combinatorial Strategies Against Glioblastoma
Source: Front Immunol. 2020 Sep 29;11:571951. doi: 10.3389/fimmu.2020.571951 (PMC7552736; doi:10.3389/fimmu.2020.571951)
Supplement: Supplementary file 1 [file Table_1.docx]

***Supplementary table 1: Clinical trials of immunotherapies in GBM and glioma***

| Type | Target | Treatment | Title | Phase | Clinical trial # |
| --- | --- | --- | --- | --- | --- |
| Checkpoint inhibitor | PD-1 | Nivolumab | Neoadjuvant Nivolumab in Glioblastoma (Neo-nivo) (Completed) | 2 | NCT02550249 |
|  |  | Pembrolizumab  CART-EGFRvIII T cells | Phase 1 Study of EGFRvIII-Directed CAR T Cells Combined With PD-1 Inhibition in Patients with Newly Diagnosed, MGMT Unmethylated Glioblastoma. | 1 | NCT03726515 |
|  |  | Pembrolizumab | A Pilot Surgical Trial to Evaluate Early Immunologic Pharmacodynamic Parameters For The PD-1 Checkpoint Inhibitor, Pembrolizumab (MK-3475), In Patients With Surgically Accessible Recurrent/Progressive Glioblastoma | 1 | NCT02852655 |
|  |  | Nivolumab  Bevacizumab | Nivolumab With Radiation Therapy and Bevacizumab for Recurrent MGMT Methylated Glioblastoma | 2 | NCT03743662 |
|  |  | TIL expressing PD-1 antibody | Tumor-infiltrating T Lymphocyte (TIL) Adoptive Therapy for Patients With Glioblastoma Multiforme | 1 | NCT03347097 |
|  |  | INT230-6 (cell permeation enhancer, cisplatin and vinblastine sulfate)  Anti-PD-1 antibody | A Phase 1/2 Safety Study of Intratumorally Administered INT230-6 in Adult Subjects with Advanced Refractory Cancers | 1/2 | NCT03058289 |
|  |  | Nivolumab  Varlilumab | A Dose Escalation and Cohort Expansion Study of Anti-CD27 (Varlilumab) and Anti-PD-1 (Nivolumab) in Advanced Refractory Solid Tumors (Completed) | 1/2 | NCT02335918 |
|  |  | Pembrolizumab added to TMZ/RT | Pembrolizumab for Newly Diagnosed Glioblastoma (PERGOLA) | 2 | NCT03899857 |
|  |  | Nivolumab | Nivolumab in People with IDH-Mutant Gliomas with and without Hypermutator Phenotype | 2 | NCT03718767 |
|  |  | Pembrolizumab  Lenvatinib | Efficacy and Safety of Pembrolizumab (MK-3475) Plus Lenvatinib (E7080/MK-7902) in Previously Treated Participants With Select Solid Tumors (MK-7902-005/E7080-G000-224/LEAP-005) | 2 | NCT03797326 |
|  |  | Nivolumab  Bevacizumab | Translational Study of Nivolumab in Combination with Bevacizumab for Recurrent Glioblastoma | 2 | NCT03890952 |
|  |  | TTAC-0001 (anti-VEGFR-2/KDR)  Pembrolizumab | TTAC-0001 and Pembrolizumab Combination phase1b Trial in Recurrent Glioblastoma | 1 | NCT03722342 |
|  | PD-L1 | Anti-PD-L1 chimeric switch receptor (CSR) T cells | Pilot Study of Autologous Chimeric Switch Receptor Modified T Cells in Recurrent Glioblastoma Multiforme (Unknown Status) | 1 | NCT02937844 |
|  |  | Durvalumab  Hypofractionated stereotactic RT | A Study Evaluating the Association of Hypofractionated Stereotactic Radiation Therapy and Durvalumab for Patients with Recurrent Glioblastoma (STERIMGLI) | 1/2 | NCT02866747 |
|  |  | Avelumab  Hypofractionated RT (HFRT) | Avelumab with Hypofractionated Radiation Therapy in Adults With Isocitrate Dehydrogenase (IDH) Mutant Glioblastoma | 2 | NCT02968940 |
|  |  | Avelumab  MRI-guided Laser Interstitial Thermal Therapy (LITT) | Avelumab with Laser Interstitial Therapy for Recurrent Glioblastoma | 1 | NCT03341806 |
|  |  | MEDI4736 (Durvalumab)  RT  Bevacizumab | Phase 2 Study of MEDI4736 in Patients with Glioblastoma | 2 | NCT02336165 |
|  |  | Axitinbib (VEGFR-specific TKI)  Avelumab | Clinical Trial on the Combination of Avelumab and Axitinib for the Treatment of Patients with Recurrent Glioblastoma (GliAvAx) (Completed) | 2 | NCT03291314 |
|  |  | Avelumab | Avelumab in Patients with Newly Diagnosed Glioblastoma Multiforme (SEJ) | 2 | NCT03047473 |
|  | CTLA-4 | Ipilimumab  Nivolumab | Intra-tumoral Ipilimumab Plus Intravenous Nivolumab Following the Resection of Recurrent Glioblastoma (GlitIpNi) | 1 | NCT03233152 |
|  |  | Nivolumab  Anti-GITR mAb (MK-4166)  IDO1 Inhibitor (INCB024360)  Ipilimumab | Biomarker-Driven Therapy Using Immune Activators With Nivolumab in Patients With First Recurrence of Glioblastoma | 1 | NCT03707457 |
|  |  | Tremelimumab  Durvalumab | Tremelimumab and Durvalumab in Combination or Alone in Treating Patients With Recurrent Malignant Glioma | 2 | NCT02794883 |
|  |  | Ipilimumab  Nivolumab  TMZ | Ipilimumab and/or Nivolumab in Combination With Temozolomide in Treating Patients With Newly Diagnosed Glioblastoma or Gliosarcoma | 1 | NCT02311920 |
|  | LAG-3 | BMS-986016 (anti-LAG-3 mAb)  Nivolumab  Urelumab (anti-CD137) | Anti-LAG-3 Alone & in Combination w/ Nivolumab Treating Patients w/ Recurrent GBM (Anti-CD137 Arm Closed 10/16/18) | 1 | NCT02658981 |
|  |  | BMS-986016 (anti-LAG-3 mAb)  Nivolumab | Cytokine Microdialysis for Real-Time Immune Monitoring in Glioblastoma Patients Undergoing Checkpoint Blockade | 1 | NCT03493932 |
|  | TIM-3 | Anti-TIM3 mAb (MBG453)  Spartalizumab  stereotactic radiosurgery (SRS) | Trial of Anti-Tim-3 in Combination With Anti-PD-1 and SRS in Recurrent GBM | 1 | NCT03961971 |
| DC vaccine | | DC vaccine  allogeneic hematopoietic stem cells  cytotoxic lymphocytes | Proteome-based Personalized Immunotherapy of Glioblastoma | 2/3 | NCT01759810 |
|  |  | ICT-121 DC vaccine (CD133 pulsed) | A Study of ICT-121 Dendritic Cell Vaccine in Recurrent Glioblastoma  (Completed) | 1 | NCT02049489 |
|  |  | Tumor lysate DC vaccine | Efficiency of Vaccination With Lysate-loaded Dendritic Cells in Patients With Newly Diagnosed Glioblastoma (GlioVax) | 2 | NCT03395587 |
|  |  | Tumor lysate DC vaccine  TMZ/RT | Phase II Feasibility Study of Dendritic Cell Vaccination for Newly Diagnosed Glioblastoma Multiforme (Completed) | 2 | NCT00323115 |
|  |  | Tumor lysate DC vaccine | Efficacy & Safety of Autologous Dendritic Cell Vaccination in Glioblastoma Multiforme After Complete Surgical Resection  (Completed) | 2 | NCT01006044 |
|  |  | Wilms' tumor 1 (WT1) messenger (m)RNA-loaded DC vaccination  TMZ/RT | Adjuvant Dendritic Cell-immunotherapy Plus Temozolomide in Glioblastoma Patients (ADDIT-GLIO) | 1/2 | NCT02649582 |
|  |  | Tumor lysate DC vaccine  TMZ/RT | Phase I Study of a Dendritic Cell Vaccine for Patients With Either Newly Diagnosed or Recurrent Glioblastoma | 1 | NCT02010606 |
|  |  | Tumor lysate DC vaccine | Tumor Lysate Pulsed Dendritic Cell Immunotherapy for Patients With Brain Tumors | 2 | NCT00576537 |
|  |  | Tumor lysate DC vaccine | Vaccine Therapy in Treating Patients With Recurrent Glioblastoma | 1 | NCT03360708 |
|  |  | Tumor lysate DC vaccine  TMZ | Vaccine Therapy and Temozolomide in Treating Patients With Newly Diagnosed Glioblastoma | 1 | NCT01957956 |
|  |  | DC vaccine with mRNA from tumor stem cells | Safe Study of Dendritic Cell (DC) Based Therapy Targeting Tumor Stem Cells in Glioblastoma (Completed) | 1/2 | NCT00846456 |
|  |  | Autologous tumor cell loaded DC vaccine  TMZ/RT | Dendritic Cell-Based Tumor Vaccine Adjuvant Immunotherapy of Human Glioblastoma Multiforme (WHO Grade IV Gliomas)  (Completed) | 2 | NCT02772094 |
|  |  | Cyclophosphamide (depletion of Treg)  tumor lysate DC vaccine | Autologous Dendritic Cells and Metronomic Cyclophosphamide for Relapsed High-Grade Gliomas in Children and Adolescents | 1/2 | NCT03879512 |
|  |  | Tumor lysate DC vaccine  Nivolumab | Autologous Dendritic Cells Pulsed With Tumor Lysate Antigen Vaccine and Nivolumab in Treating Patients With Recurrent Glioblastoma | 2 | NCT03014804 |
|  |  | Cytomegalovirus (CMV) mRNA-pulsed DC vaccine with GM-CSF  and Tetanus toxoid pre-conditioning | Cytomegalovirus (CMV) RNA-Pulsed Dendritic Cells for Pediatric Patients With Newly Diagnosed WHO Grade IV Glioma, Recurrent Malignant Glioma, or Recurrent Medulloblastoma (ATTAC-P) | 1 | NCT03615404 |
|  |  | Glioma stem-like cells loaded DC vaccine  TMZ/RT | Study of DC Vaccination Against Glioblastoma | 2 | NCT01567202 |
|  |  | DC vaccine transfected with mRNA from autologous tumor stem cells, Survivin and hTERT  TMZ | Dendritic Cell Immunotherapy Against Cancer Stem Cells in Glioblastoma Patients Receiving Standard Therapy (DEN-STEM) | 2/3 | NCT03548571 |
|  |  | Tumor lysate DC vaccine + Trivax  TMZ/RT | Dendritic Cell Cancer Vaccine for High-grade Glioma (GBM-Vax)  (Completed) | 2 | NCT01213407 |
|  |  | TAA pulsed DC vaccine | Immunotherapy for Patients With Brain Stem Glioma and Glioblastoma (Completed) | 1 | NCT00576641 |
|  |  | CMV mRNA pulsed DC vaccine  with Tetanus toxoid pre-conditioning  Basiliximab  TMZ | DC Migration Study for Newly-Diagnosed GBM (ELEVATE) | 2 | NCT02366728 |
|  |  | CMV mRNA pulsed DC vaccine with Tetanus toxoid pre-conditioning  Therapeutic autologous lymphocytes | Vaccine Therapy in Treating Patients With Newly Diagnosed Glioblastoma Multiforme (ATTAC) | 1 | NCT00639639 |
|  |  | CMV mRNA pulsed DC vaccine with GM-CSF and Tetanus toxoid pre-conditioning | Vaccine Therapy for the Treatment of Newly Diagnosed Glioblastoma Multiforme (ATTAC-II) | 2 | NCT02465268 |
|  |  | CMV mRNA-pulsed DC vaccine with GM-CSF and Tetanus toxoid pre-conditioning  TMZ  111-Indium-labeled DC (in vivo trafficking studies) | Immunotherapy Targeted Against Cytomegalovirus in Patients With Newly-Diagnosed WHO Grade IV Unmethylated Glioma (I-ATTAC) | 2 | NCT03927222 |
|  |  | CMV mRNA pulsed DC vaccine with Tetanus toxoid pre-conditioning  111-Indium-labeled CMV pulsed DC  HIV-Gag mRNA pulsed DC  TMZ  Varlilumab | DC Migration Study to Evaluate TReg Depletion In GBM Patients With and Without Varlilumab (DERIVe) | 2 | NCT03688178 |
|  |  | CMV pp65-LAMP mRNA-pulsed DC vaccine  Nivolumab | Nivolumab With DC Vaccines for Recurrent Brain Tumors (AVERT) | 1 | NCT02529072 |
|  |  | mRNA-TAA pulsed DC vaccine  allogeneic PBMC vaccine | Personalized Cellular Vaccine for Glioblastoma (PERCELLVAC) | 1/2 | NCT02709616 |
|  |  | WT1 mRNA-transfected DC vaccine | Dendritic Cell Vaccination for Patients With Solid Tumors (Unknown Status) | 1/2 | NCT01291420 |
|  |  | TAA-pulsed DC vaccine with prior Cyclophosphamide and Bevacizumab afterwards | Immune Modulatory DC Vaccine Against Brain Tumor | 1 | NCT03914768 |
|  |  | GBM stem-like cells (GSC) loaded DC vaccine | A Phase I Study of Immunotherapy With GSC -Loaded Dendritic Cells in Patients With Recurrent Glioblastoma (DENDR-STEM) (unknown status) | 1 | NCT02820584 |
|  |  | GSC loaded DC vaccine | Vaccination With Dendritic Cells Loaded With Brain Tumor Stem Cells for Progressive Malignant Brain Tumor | 1 | NCT01171469 |
|  |  | Autologous TAA pulsed DC vaccine and GM-CSF | Autologous Dendritic Cells Loaded With Autologous Tumor Associated Antigens for Treatment of Newly Diagnosed Glioblastoma | 2 | NCT03400917 |
|  |  | CMV mRNA-pulsed DC vaccine with GM-CSF  Basiliximab | Basiliximab in Treating Patients With Newly Diagnosed Glioblastoma Multiforme Undergoing Targeted Immunotherapy and Temozolomide-Caused Lymphopenia (REGULATe) | 1 | NCT00626483 |
|  |  | Tumor lysate pulsed DC vaccine | Expanded Access Protocol for GBM Patients With Already Manufactured DCVax®-L Who Have Screen-Failed Protocol 020221 (DCVax-L EAP) | - | NCT02146066 |
|  |  | GSC-mRNA loaded DC vaccine | Vaccine Therapy in Treating Patients Undergoing Surgery for Recurrent Glioblastoma Multiforme (Completed) | 1 | NCT00890032 |
|  |  | Cancer-testis antigen (NY-ESO-1) pulsed DC vaccine  Sirolimus | Vaccine Therapy With or Without Sirolimus in Treating Patients With NY-ESO-1 Expressing Solid Tumors (Completed) | 1 | NCT01522820 |
|  |  | TAA pulsed DC vaccine | Vaccine Therapy in Treating Patients With Malignant Glioma (Completed) | 1 | NCT00612001 |
|  |  | Tumor lysate-pulsed DC vaccine | Vaccine Therapy in Treating Patients With Malignant Glioma  (Completed) | 1 | NCT00068510 |
|  |  | Autologous TAA pulsed DC vaccine (ICT-107) | Phase 3 Randomized, Double-blind, Controlled Study of ICT-107 in Glioblastoma (Suspended) | 3 | NCT02546102 |
|  |  | Autologous TAA pulsed DC vaccine (ICT-107) | A Study of ICT-107 Immunotherapy in Glioblastoma Multiforme (GBM) (Completed) | 2 | NCT01280552 |
| Peptide vaccine | | Cytomegalovirus peptide (PEP-CMV)  TMZ | Peptide Targets for Glioblastoma Against Novel Cytomegalovirus Antigens (PERFORMANCE) | 1 | NCT02864368 |
|  |  | IMA950 multi-peptide vaccine  GM-CSF  TMZ/RT | Vaccine Therapy, Temozolomide, and Radiation Therapy in Treating Patients With Newly Diagnosed Glioblastoma Multiforme (Completed) | 1 | NCT01222221 |
|  |  | Heat shock protein peptide complex (HSPPC-96)  TMZ | HSPPC-96 Vaccine With Temozolomide in Patients With Newly Diagnosed GBM (HeatShock) (Completed) | 2 | NCT00905060 |
|  |  | Heat shock protein peptide complex (HSPPC-96) | Research for Immunotherapy of Glioblastoma With Autologous Heat Shock Protein gp96 (Completed) | 1 | NCT02122822 |
|  |  | Heat shock protein peptide complex (HSPPC-96) | GP96 Heat Shock Protein-Peptide Complex Vaccine in Treating Patients With Recurrent or Progressive Glioma (Completed) | 1/2 | NCT00293423 |
|  |  | Heat shock protein peptide complex (HSPPC-96)  Bevacizumab | Vaccine Therapy With Bevacizumab Versus Bevacizumab Alone in Treating Patients With Recurrent Glioblastoma Multiforme That Can Be Removed by Surgery | 2 | NCT01814813 |
|  |  | Heat shock protein peptide complex (HSPPC-96)  RT | Trial of Heat Shock Protein Peptide Complex-96 (HSPPC-96) Vaccine | 1 | NCT02722512 |
|  |  | SVN53-67/M57-keyhole limpet hemocyanin (KLH) peptide vaccine (SurVaxM)  Montanide ISA 51 VG  GM-CSF  TMZ | SurVaxM Vaccine Therapy and Temozolomide in Treating Patients With Newly Diagnosed Glioblastoma | 2 | NCT02455557 |
|  |  | Pembrolizumab  SurVaxM  GM-CSF  Montanide ISA 51 VG | Phase II Study of Pembrolizumab Plus SurVaxM for Glioblastoma at First Recurrence | 2 | NCT04013672 |
|  |  | PEP-3-KLH conjugate vaccine  Basiliximab/Daclizumab  TMZ/RT | Chemotherapy, Radiation Therapy, and Vaccine Therapy With Basiliximab in Treating Patients With Glioblastoma Multiforme That Has Been Removed by Surgery (ZAP IT) (Completed) | 1 | NCT00626015 |
|  |  | PEP-3-KLH conjugate vaccine  GM-CSF  TMZ | Vaccine Therapy in Treating Patients With Newly Diagnosed Glioblastoma Multiforme (ACTIVATe) (Completed) | 2 | NCT00643097 |
|  |  | Montanide ISA-51/Survivin Peptide Vaccine  GM-CSF | Vaccine Therapy and Sargramostim in Treating Patients With Malignant Glioma (Completed) | 1 | NCT01250470 |
|  |  | WT1 protein-derived peptide vaccine DSP-7888 | A Study of DSP-7888 in Pediatric Patients With Relapsed or Refractory High Grade Gliomas | 1/2 | NCT02750891 |
|  |  | Telomerase: 540-548 peptide vaccine  GM-CSF | Vaccine Therapy and Sargramostim in Treating Patients With Sarcoma or Brain Tumor (Completed) | 1 | NCT00069940 |
| DNA vaccine | | VEGFR-2 DNA vaccine (VXM01) | VXM01 Phase I Pilot Study in Patients With Operable Recurrence of a Glioblastoma (Completed) | 1 | NCT02718443 |
|  |  | VXM01  Avelumab | VXM01 Plus Avelumab Combination Study in Progressive Glioblastoma | 1/2 | NCT03750071 |
|  |  | INO-5401 (3 separate DNA plasmids targeting Wilms tumor gene-1 (WT1) antigen, prostate-specific membrane antigen (PSMA) and human telomerase reverse transcriptase (hTERT) genes  INO-9012 (DNA plasmid for expression of human interleukin-12)  Cemiplimab  TMZ/RT | INO-5401 and INO-9012 Delivered by Electroporation (EP) in Combination with Cemiplimab (REGN2810) in Newly-Diagnosed Glioblastoma (GBM) | 1/2 | NCT03491683 |
| Gene-mediated cytotoxic immunotherapy | | AdV-tk/GCV | ADV-TK Improves Outcome of Recurrent High-Grade Glioma (HGG-01) (Completed) | 2 | NCT00870181 |
|  |  | AdV-tk/VCV  RT | Phase 1b Study of AdV-tk + Valacyclovir Combined With Radiation Therapy for Malignant Gliomas (BrTK01) (Completed) | 1 | NCT00751270 |
|  |  | AdV-tk/VCV  RT | Phase 2a Study of AdV-tk With Standard Radiation Therapy for Malignant Glioma (BrTK02) (Completed) | 2 | NCT00589875 |
|  |  | AdV-tk/VCV  Nivolumab  TMZ/RT | Gene Mediated Cytotoxic Immunotherapy (GMCI), Nivolumab, and Radiation Therapy in Treating Patients with Newly Diagnosed High-Grade Gliomas (GMCI) | 1 | NCT03576612 |
|  |  | AdV-tk/VCV  RT | A Phase I Study of AdV-tk + Prodrug Therapy in Combination With Radiation Therapy for Pediatric Brain Tumors | 1 | NCT00634231 |
| Oncolytic virus | | TG6002 (oncolytic virus)  5-flucytosine | Safety and Efficacy of the ONCOlytic VIRus Armed for Local Chemotherapy, TG6002/5-FC, in Recurrent Glioblastoma Patients (ONCOVIRAC) | 1/2 | NCT03294486 |
|  |  | DNX-2440 | DNX-2440 Oncolytic Adenovirus for Recurrent Glioblastoma | 1 | NCT03714334 |
|  |  | New Castle Disease Virus | New Castle Disease Virus (NDV) in Glioblastoma Multiforme (GBM), Sarcoma and Neuroblastoma (Withdrawn) | 1 | NCT01174537 |
|  |  | DNX-2401  Interferon-gamma | DNX-2401 With Interferon Gamma (IFN-γ) for Recurrent Glioblastoma or Gliosarcoma Brain Tumors (TARGET-I) (Completed) | 1 | NCT02197169 |
|  |  | Parvovirus H-1 | Parvovirus H-1 (ParvOryx) in Patients With Progressive Primary or Recurrent Glioblastoma Multiforme. (ParvOryx01) (Completed) | 1/2 | NCT01301430 |
|  |  | G207 | Safety and Effectiveness Study of G207, a Tumor-Killing Virus, in Patients With Recurrent Brain Cancer (Completed) | 1/2 | NCT00028158 |
|  |  | rQNestin34.5v.2  Cyclophosphamide | A Study of the Treatment of Recurrent Malignant Glioma With rQNestin34.5v.2 (rQNestin) | 1 | NCT03152318 |
|  |  | M032 | Genetically Engineered HSV-1 Phase 1 Study for the Treatment of Recurrent Malignant Glioma (M032-HSV-1) | 1 | NCT02062827 |
|  |  | DNX-2401  Pembrolizumab | Combination Adenovirus + Pembrolizumab to Trigger Immune Virus Effects (CAPTIVE) | 2 | NCT02798406 |
|  |  | C134 | Trial of C134 in Patients With Recurrent GBM (C134-HSV-1) | 1 | NCT03657576 |
|  |  | DNX-2401  TMZ | Virus DNX2401 and Temozolomide in Recurrent Glioblastoma (D24GBM) (Completed) | 1 | NCT01956734 |
|  |  | G207 | HSV G207 in Children With Recurrent or Refractory Cerebellar Brain Tumors | 1 | NCT03911388 |
|  |  | G207 | HSV G207 Alone or With a Single Radiation Dose in Children With Progressive or Recurrent Supratentorial Brain Tumors | 1 | NCT02457845 |
|  |  | REOLYSIN® therapeutic reovirus | Safety and Efficacy Study of REOLYSIN® in the Treatment of Recurrent Malignant Gliomas (Completed) | 1 | NCT00528684 |
|  |  | HSV-1716  Dexamethasone | Oncolytic HSV-1716 in Treating Younger Patients With Refractory or Recurrent High Grade Glioma That Can Be Removed By Surgery (Terminated) | 1 | NCT02031965 |
|  |  | DNX-2401 | Oncolytic Adenovirus DNX-2401 in Treating Patients With Recurrent High-Grade Glioma | 1 | NCT03896568 |
|  |  | NSC-CRAd-Survivin-pk7  (Neural stem cells loaded with an oncolytic adenovirus) | Neural Stem Cell Based Virotherapy of Newly Diagnosed Malignant Glioma | 1 | NCT03072134 |
|  |  | PVSRIPO (Polio/Rhinovirus Recombinant | Phase 1b Study PVSRIPO for Recurrent Malignant Glioma in Children | 1 | NCT03043391 |
| PRR | | Bevacizumab  Poly-ICLC (TLR3 agonist)  Tumor associated long synthetic peptide Vaccine | A Toll-like Receptor Agonist as an Adjuvant to Tumor Associated Antigens (TAA) Mixed With Montanide ISA-51 VG With Bevacizumab for Patients With Recurrent Glioblastoma | 2 | NCT02754362 |
|  |  | Tumor lysate-pulsed DC vaccination  Resiquimod (TLR7/8 agonist) or  Poly-ICLC (TLR3 agonist) | Dendritic Cell Vaccine for Patients With Brain Tumors | 2 | NCT01204684 |
|  |  | DC Vaccine  post-DC vaccine administration of tumor lysate + Imiquimod (TLR7/8 agonist) | Dendritic Cell (DC) Vaccine for Malignant Glioma and Glioblastoma | 1 | NCT01808820 |
|  |  | IMA950 multi-peptide vaccine  GM-CSF  Imiquimod | Peptide-based Glioma Vaccine IMA950 in Patients With Glioblastoma (Terminated) | 1 | NCT01403285 |
|  |  | poly I:C  GM-CSF  RT | Combination of Immunization and Radiotherapy for Recurrent GBM (InSituVac1) | 1 | NCT03392545 |
|  |  | IMA950 multi-peptide vaccine  Poly-ICLC | Phase I/II Trial of IMA950 Multi-peptide Vaccine Plus Poly-ICLC in Glioblastoma (Completed) | 1/2 | NCT01920191 |
|  |  | Actively personalized vaccination (APVAC)  Poly-ICLC  GM-CSF | GAPVAC Phase I Trial in Newly Diagnosed Glioblastoma Patients (Completed) | 1 | NCT02149225 |
|  |  | Personalized peptide vaccine  Poly-ICLC  TMZ | Neoepitope-based Personalized Vaccine Approach in Patients With Newly Diagnosed Glioblastoma (Terminated) | 1 | NCT02510950 |
|  |  | Mutation-derived tumor antigen vaccine  Poly-ICLC  Tumor Treating Fields | Safety and Immunogenicity of Personalized Genomic Vaccine and Tumor Treating Fields (TTFields) to Treat Glioblastoma | 1 | NCT03223103 |
|  |  | Personalized neoantigen-based vaccine + Poly-ICLC (NeoVax)  Nivolumab  Ipilimumab | Neoantigen-based Personalized Vaccine Combined With Immune Checkpoint Blockade Therapy in Patients With Newly Diagnosed, Unmethylated Glioblastoma | 1 | NCT03422094 |
|  |  | NeoVax  Pembrolizumab  RT | Personalized NeoAntigen Cancer Vaccine w RT Plus Pembrolizumab for Patients With MGMT Unmethylated, Newly Diagnosed GBM | 1 | NCT02287428 |
| Adoptive cell therapy | EGFRvIII | Anti-EGFRvIII CAR T cells  Cyclophosphamide  Fludarabine | Pilot Study of Autologous Anti-EGFRvIII CAR T Cells in Recurrent Glioblastoma Multiforme | 1 | NCT02844062 |
|  |  | CART-EGFRvIII T cells  Pembrolizumab | CART-EGFRvIII + Pembrolizumab in GBM | 1 | NCT03726515 |
|  |  | EGFRvIII CAR T cells | EGFRvIII CAR T Cells for Newly-Diagnosed WHO Grade IV Malignant Glioma (ExCeL) | 1 | NCT02664363 |
|  |  | Epidermal growth factor receptor(EGFRv)III Chimeric antigen receptor (CAR) transduced PBL  Aldesleukin  Fludarabine  Cyclophosphamide | CAR T Cell Receptor Immunotherapy Targeting EGFRvIII for Patients With Malignant Gliomas Expressing EGFRvIII | 1/2 | NCT01454596 |
|  |  | EGFRvIII-CARs | Intracerebral EGFR-vIII CAR-T Cells for Recurrent GBM (INTERCEPT) | 1 | NCT03283631 |
|  |  | Chimeric antigen receptor T cells | Personalized Chimeric Antigen Receptor T Cell Immunotherapy for Patients With Recurrent Malignant Gliomas | 1 | NCT03423992 |
|  |  | CART-EGFRvIII T cells | Autologous T Cells Redirected to EGFRVIII-With a Chimeric Antigen Receptor in Patients With EGFRVIII+ Glioblastoma | 1 | NCT02209376 |
|  |  | CAR-T/TCR-T cells immunotherapy | Autologous CAR-T/TCR-T Cell Immunotherapy for Solid Malignancies | 1/2 | NCT03941626 |
|  |  | CAR-T cell immunotherapy | Autologous CAR-T/TCR-T Cell Immunotherapy for Malignancies | 1/2 | NCT03638206 |
|  | ERBB2/  HER-2 | HER.CAR CMV-specific CTLs | CMV-specific Cytotoxic T Lymphocytes Expressing CAR Targeting HER2 in Patients With GBM (HERT-GBM) | 1 | NCT01109095 |
|  |  | CD19CAR-CD28-CD3zeta-EGFRt-expressing Tcm-enriched T-lymphocytes  CD19CAR-CD28-CD3zeta-EGFRt-expressing Tn/mem-enriched T-lymphocytes | Memory-Enriched T Cells in Treating Patients With Recurrent or Refractory Grade III-IV Glioma | 1 | NCT03389230 |
|  |  | Chimeric antigen receptor T cells | Personalized Chimeric Antigen Receptor T Cell Immunotherapy for Patients With Recurrent Malignant Gliomas | 1 | NCT03423992 |
|  |  | Anti-HER2 CAR-T | A Clinical Research of CAR T Cells Targeting HER2 Positive Cancer | 1/2 | NCT02713984 |
|  |  | HER2-specific chimeric antigen receptor (CAR) T cell | HER2-specific CAR T Cell Locoregional Immunotherapy for HER2-positive Recurrent/Refractory Pediatric CNS Tumors | 1 | NCT03500991 |
|  |  | NK-92/5.28.z | Intracranial Injection of NK-92/5.28.z Cells in Patients With Recurrent HER2-positive Glioblastoma (CAR2BRAIN) | 1 | NCT03383978 |
|  | GD2 | Chimeric antigen receptor T cells | Personalized Chimeric Antigen Receptor T Cell Immunotherapy for Patients With Recurrent Malignant Gliomas | 1 | NCT03423992 |
|  |  | (C7R)-GD2.CART cells  Cyclophosphamide  Fludarabine | C7R-GD2.CAR T Cells for Patients With GD2-expressing Brain Tumors (GAIL-B) | 1 | NCT04099797 |
|  |  | CAR-T cell immunotherapy | CAR-T Cell Immunotherapy for GD2 Positive Glioma Patients | 1/2 | NCT03252171 |
|  |  | GD2 CAR T cells  Fludarabine  Cyclophosphamide | GD2 CAR T Cells in Diffuse Intrinsic Pontine Gliomas(DIPG) & Spinal Diffuse Midline Glioma(DMG) | 1 | NCT04196413 |
|  | IL13RA2 | IL13Ralpha2-specific Hinge-optimized 4-1BB-co-stimulatory CAR/Truncated CD19-expressing Autologous TN/MEM Cells  IL13Ralpha2-specific Hinge-optimized 41BB-co-stimulatory CAR Truncated CD19-expressing Autologous T-Lymphocytes | Genetically Modified T-cells in Treating Patients With Recurrent or Refractory Malignant Glioma | 1 | NCT02208362 |
|  |  | IL13Ralpha2-specific Hinge-optimized 4-1BB-co-stimulatory CAR/Truncated CD19-expressing Autologous TN/MEM Cells  Ipilimumab  Nivolumab | IL13Ralpha2-Targeted Chimeric Antigen Receptor (CAR) T Cells With or Without Nivolumab and Ipilimumab in Treating Patients With Recurrent or Refractory Glioblastoma | 1 | NCT04003649 |
|  |  | Chimeric antigen receptor T cells | Personalized Chimeric Antigen Receptor T Cell Immunotherapy for Patients With Recurrent Malignant Gliomas | 1 | NCT03423992 |
|  | CD276/  B7-H3 | TMZ  B7-H3 CAR-T | B7-H3 CAR-T for Recurrent or Refractory Glioblastoma | 1/2 | NCT04077866 |
|  |  | SCRI-CARB7H3(s); B7H3-specific chimeric antigen receptor (CAR) T cells | Study of B7-H3-Specific CAR T Cell Locoregional Immunotherapy for Diffuse Intrinsic Pontine Glioma/Diffuse Midline Glioma and Recurrent or Refractory Pediatric Central Nervous System Tumors | 1 | NCT04185038 |
|  | EGFR | Anti-EGFR CAR T | CAR T Cells in Treating Patients With Malignant Gliomas Overexpressing EGFR | 1 | NCT02331693 |
|  |  | EGFR806-specific chimeric antigen receptor (CAR) T cell | EGFR806-specific CAR T Cell Locoregional Immunotherapy for EGFR-positive Recurrent or Refractory Pediatric CNS Tumors | 1 | NCT03638167 |
|  | EPHA2 | Chimeric antigen receptor T cells | Personalized Chimeric Antigen Receptor T Cell Immunotherapy for Patients With Recurrent Malignant Gliomas | 1 | NCT03423992 |
|  |  | CAR-T cell immunotherapy | CAR-T Cell Immunotherapy for EphA2 Positive Malignant Glioma Patients | 1/2 | NCT02575261 |
|  | MUC1 | Anti-MUC1 CAR-T cells | CAR-T Cell Immunotherapy in MUC1 Positive Solid Tumor | 1/2 | NCT02617134 |
|  |  | Anti-MUC1 CAR-pNK cells | CAR-pNK Cell Immunotherapy in MUC1 Positive Relapsed or Refractory Solid Tumor | 1/2 | NCT02839954 |
|  | PROM1/  CD133 | Chimeric antigen receptor T cells | Personalized Chimeric Antigen Receptor T Cell Immunotherapy for Patients With Recurrent Malignant Gliomas | 1 | NCT03423992 |
|  | BSG/  CD147 | CD147-CART | CD147-CART Cells in Patients With Recurrent Malignant Glioma | 1 | NCT04045847 |
|  | Chlorotoxin | Chlorotoxin (EQ)-CD28-CD3zeta-CD19t-expressing CAR T-lymphocytes | Chimeric Antigen Receptor (CAR) T Cells With a Chlorotoxin Tumor-Targeting Domain for the Treatment of MPP2+ Recurrent or Progressive Glioblastoma | 1 | NCT04214392 |
|  | KLRK1/  NKG2D | NKG2D-based CAR T-cells | NKG2D-based CAR T-cells Immunotherapy for Patient With r/r NKG2DL+ Solid Tumors | 1 | NCT04270461 |
|  | CD274/  PD-L1 | Anti-PD-L1 CSR T cells  Cyclophosphamide  Fludarabine | Pilot Study of Autologous Chimeric Switch Receptor Modified T Cells in Recurrent Glioblastoma Multiforme | 1 | NCT02937844 |
