# Supplementary material for: Microglia-Centered Combinatorial Strategies Against Glioblastoma
Source: Front Immunol. 2020 Sep 29;11:571951. doi: 10.3389/fimmu.2020.571951 (PMC7552736; doi:10.3389/fimmu.2020.571951)
Supplement: Supplementary file 2 [file Data_Sheet_1.docx]

**Supplementary information**

**Overview of current immunotherapies against GBM**

Here, we summarize the most recent clinical trials and their strategies in interfering with the innate and adaptive GBM-iTME.

***Antibody-based immune checkpoint inhibition***

The first phase III clinical trial of an immune checkpoint inhibitor against GBM started in 2014 ([NCT02017717](https://clinicaltrials.gov/ct2/show/NCT02017717)). Its purpose was to assess the safety and efficacy of nivolumab (anti-PDCD1 or PD1) alone vs. bevacizumab (anti-VEGFA) and in combination with ipilimumab (anti-CTLA4). This trial demonstrated that nivolumab is better tolerated in monotherapy than in combination with ipilimumab and that the tolerability of their combination is dose-dependent in regard to ipilimumab ([1](#_ENREF_1)). Another large phase III clinical trial for the combination of nivolumab and radiotherapy vs. TMZ and radiotherapy in GBM is underway since 2016 ([NCT02617589](https://clinicaltrials.gov/ct2/show/NCT02617589)). It has been recently shown that programmed cell death 1 (PDCD1)/CD274 or PDL1 blockade as neoadjuvant therapy in patients with recurrent GBM confers significant clinical benefit with increased overall and progression-free survival. This is associated with the expression of chemokine transcripts, T cell, and interferon gamma (*IFNG*)-related genes that consequently increase immune cell infiltration and expand T cell receptor (TCR) clonal diversity ([2](#_ENREF_2), [3](#_ENREF_3)). Another study assessed the correlation between genomic mutations and anti-PDCD1 clinical responses, showing that the enrichment of *PTEN* mutations is associated with the expression of an immunosuppressive signature in non-responders and an enrichment of MAPK pathway alterations in responders to anti-PDCD1 therapy ([4](#_ENREF_4)).

***Dendritic cell-mediated and personalized cancer vaccination therapy***

Dendritic cells (DCs) are the most potent specialized APCs that uptake pathogens/antigens and process them for cross-presentation to either CD4^+^ T-helper cells, by loading them on MHC class II molecules, or CD8^+^ cytotoxic T cells, on MHC class I molecules ([5](#_ENREF_5)). One strategy in active cancer immunotherapy is to mature the patient’s own DCs *in vitro*, load them with tumor antigens and reinfuse them to the patient leading to T cell activation. Preparation of mature DCs can be achieved by transfection of naive DCs with total protein contents or defined peptides isolated from tumor cells; DNA, RNA or viral transfection/transduction; or DC-tumor cell fusions ([6-9](#_ENREF_6)). Several DC vaccination preclinical and clinical studies have been reported against primary and recurrent GBM (Supplementary Table 1).

***Oncolytic virus-mediated therapy***

Oncolytic viruses can target, replicate, and destroy cancer cells. Cancer cells are excellent hosts for viral infection and replication since their protein synthesis machinery is permanently activated and their apoptotic pathways are often impaired. The oncolytic activity of most oncolytic viruses does not only act through direct lysis of cancer cells, but also by the induction of systemic immune responses leading to cell death and release of damage-associated molecular patterns (DAMPs) and tumor-associated antigens (TAAs) from virus-infected cells. They also release viral pathogen-associated molecular patterns (PAMPs) contributing to APC maturation that results in the activation of antigen-specific CD4^+^ and CD8^+^ T cell responses. Once activated, CD8^+^ T cells traffic to tumor sites, where they become cytotoxic to tumor cells upon antigen recognition ([10](#_ENREF_10)). The oncolytic properties of many viruses have been studied in preclinical models for their potential antitumor effects. The first oncolytic virotherapy for GBM was reported in 1991, where a thymidine kinase negative mutant of herpes simplex virus type 1 (HSV-1) attenuated for neurovirulence caused growth inhibition of a U87 human glioma mouse xenograft and prolonged host survival ([11](#_ENREF_11)). Since then, a multitude of other oncolytic HSV mutants have been generated. These modified strains exhibit improved features such as safety, reduced [neurotoxicity](https://www.omicsonline.org/open-access/bilirubininduced-neurotoxicity-and-environmental-impacts-onhyperbilirubinemia-development-2161-0525-1000414.php?aid=82531), conditional tumor-selective replication, and high titer viral production. Some of these mutants are currently being tested in clinical trials, making HSV-1 the most extensively studied oncolytic virus in glioma therapy ([12](#_ENREF_12)). Adenoviruses have also been extensively studied for their potential oncolytic properties. For instance, ONYX-015, an adenovirus mutant, is thought to replicate more efficiently in cells with disrupted TP53 tumor suppressor pathway. However, this mutant has been tested in a phase I clinical trial for glioma patients leading to a median survival of only 6.2 months ([13](#_ENREF_13)). Despite some disappointing results, oncolytic virotherapy remains an active area of research in GBM immunotherapy (Supplementary Table 1).

***Pattern recognition receptor agonist immune modulation***

Germline-encoded pattern recognition receptors (PRRs) recognize PAMPs conserved among bacteria, viruses, fungi, and parasites, thereby activating both innate and adaptive immune cells. PRRs sense endogenous DAMPs with the resulting inflammation contributing to the healing of damaged tissue([14](#_ENREF_14)). So far, 5 classes of PRRs have been identified ([14](#_ENREF_14), [15](#_ENREF_15)). Isolated reports of prolonged survival in GBM patients developing bacterial infection proposed involvement of toll like receptors (TLRs) ([16](#_ENREF_16), [17](#_ENREF_17)). Putatively, bacterial components acting as PAMPs stimulate the immune response to target both pathogens and malignant cells. This observation has been replicated *in vivo* with stimulation of TLR4 by lipopolysaccharide (LPS) treatment, which resulted in tumor regression and prolonged survival in subcutaneous models of GBM ([18-21](#_ENREF_18)). However, the intracranial LPS stimulation of TLR4 in established tumors with a severe immunosuppressive TME failed to induce potent antitumor immune responses ([21](#_ENREF_21), [22](#_ENREF_22)). More recently, clinical studies of intracerebral injection of CpG-oligodeoxynucleotide (CpG-ODN), a TLR9 agonist, failed to improve the survival of patients with newly diagnosed GBM, despite promising preclinical studies ([22-24](#_ENREF_22)). In line with these studies, accumulating evidence points to TLR expression by tumor cells, promoting tumor cell growth, proliferation, invasion, migration, and cancer stem cell maintenance ([25](#_ENREF_25), [26](#_ENREF_26)). Underlining this, the expression of TLR2 and 9 are associated with shorter overall survival GBM patients ([27-29](#_ENREF_27)). In addition to TLR expression, there is increasing evidence supporting a protumorigenic role for some TLRs in MG-glioma crosstalk. One study reported that TLR2-mediated downregulation of MHC class II molecules in MG promotes tumor immune evasion ([30](#_ENREF_30)). Others showed that TLR4 signaling upregulates MG IL-6 secretion, which stimulates glioma growth by supporting glioma stem cells (GSCs) ([31](#_ENREF_31), [32](#_ENREF_32)). Moreover, GBM cell infiltration and expansion have been linked to TLR2-mediated upregulation of matrix metalloproteinases (MMPs) in MG ([33-35](#_ENREF_33)). However, several TLR agonists induced a survival benefit through the promotion of cell maturation, expansion, and migration of some immune cell types. TLR agonists remain under intensive clinical investigation, especially in combinatorial immunotherapy ([36](#_ENREF_36), [37](#_ENREF_37)). In fact, several ongoing clinical trials focus on the therapeutic utility of agonists, mainly those of TLR3 and 7, as adjuvants in vaccine-based cancer treatments. Our current understanding accentuates the dual role of TLRs, as their anti- or protumorigenic properties depend on tumor type, subtype of TLR, and iTME composition ([38](#_ENREF_38)).

***Bacteria-based tumor targeting***

The use of bacteria as cancer therapy dates back to the late 19^th^ century when William Coley injected *Streptococcus* into patients with inoperable cancers, leading to remission ([39](#_ENREF_39), [40](#_ENREF_40)). Many bacteria species have the potential to selectively accumulate and grow within solid tumors due to their highly hypoxic and acidic environments ([41](#_ENREF_41)). Within the tumor, some bacteria produce toxins and compete with cancer cells for nutrients ([42](#_ENREF_42)). Ultimately, the accumulation of bacteria in the tumor induces immune cell infiltration and stimulates antitumor responses, as exemplified by the treatment of bladder cancer with *Bacillus Calmette–Guérin* (BCG)-attenuated bacteria, or the use of *Salmonella* as oncolytic agents for malignant brain tumors. Genetically engineered bacteria are now generated to selectively target tumor cells, grow in the tumor environment, and deliver therapeutics locally. Therefore, engineered bacteria need guidance for selective targeting, and a system of local production and delivery of therapeutics. The challenges of trafficking and homing of bacteria to tumors have seen the development of sophisticated navigation methods such as light fields, where bacteria migrate when exposed to light; ultrasound, and magnetic fields. Once within the tumor, bacteria can be used for selective targeting of tumor cells. Recently, genetically engineered bacteria have been developed to produce a variety of cargos, including antitumor toxins, cytokines, apoptotic or autophagic agents, immunomodulators, and prodrug-converting enzymes ([41](#_ENREF_41), [43](#_ENREF_43), [44](#_ENREF_44)). Moreover, bacteria producing immune checkpoint blockers such as antagonists of CD47 and CD274 were shown to prime T cells and facilitate the clearance of cancer cells, triggering distant and durable antitumor immunity in a lymphoma model ([45](#_ENREF_45)). Another strategy relied on engineering bacteria to produce a stimulator of interferon response cGAMP interactor 1 (STING1) agonist, acting as an innate immune stimulator. Once within the tumor, the bacteria are recognized and engulfed by tumor-infiltrating APCs, where they activate the STING pathway, resulting in interferon release and tumor-specific T cell responses. An ongoing phase I clinical trial is testing the safety of this approach in refractory solid tumors (NCT04167137). Indeed, safety is a major concern for therapies with live bacteria, since robust intratumoral colonization is required for optimal therapeutic effect. Such extensive infections inevitably lead to clinical symptoms. Therefore, reasonable safety profiles with determination of dose, type of tumor to be infected, and duration of treatment must be carefully defined. Preclinical pharmacological and toxicological studies with live bacteria have shown satisfactory safety profiles in both healthy and tumor-bearing experimental animals ([46](#_ENREF_46)). However, the use of animal models for the safety and efficacy of bacterial agents is not without limitations, e.g. due to the inherent resistance of rodents to some human pathogens, rendering preclinical testing challenging ([47](#_ENREF_47)). Alternative models such as Syrian hamster or non-human primates are now favored. Although results concerning GBM-targeting bacteria are just starting to be published, this unique immunotherapy approach shows promise for the treatment of malignant brain tumors in the future.

***Nanoparticle-based iTME modulation***

Over the past decade, nanotechnology platforms have shown significant potential for modulation of the immune system in brain tumors, mainly through drug delivery systems or in combination with different therapeutic approaches, including surgery, radiotherapy, chemotherapy, and immunotherapy ([48](#_ENREF_48), [49](#_ENREF_49)). In this context, multiple reports have demonstrated that in combination with immune cells, nanoparticles significantly increased antitumor activity in GBM models ([50-52](#_ENREF_50)). However, the intrinsic impact of nanoparticles on immune cells is not well-understood. Early studies revealed a critical role for iron oxide nanoparticles in tumor growth inhibition through regulating MΦ in the iTME of solid tumors; a phenomenon that is thought to result from proinflammatory Th1-type responses ([53](#_ENREF_53)). Future research must determine the most effective methods of targeting the iTME of brain tumors through innate and adaptive immune cell subsets by investigating the behavioral interactions between nanomaterials and biological processes.

***Intratumoral cytokine delivery***

Various proinflammatory cytokines have been administered locally to counteract the anti-inflammatory and immunosuppressive GBM-iTME and to increase the numbers of tumor-reactive effector cells. IL-12 is a potent inducer of antitumor immune responses, mainly via IFNG, but highly toxic when applied systemically ([54](#_ENREF_54)). Intratumoral IL-12 infusion repolarizes the GBM-iTME and boosts systemically-applied checkpoint blockade ([55](#_ENREF_55)). Compared to preclinical studies of IL-2 and IFNB1, mechanistic functional analysis in the context of local GBM therapy revealed that CD4^+^ and CD8^+^ T cells, but not NK cells are crucial effector cells. Switching from intratumoral delivery via osmotic minipumps to oncolytic, IL-12-expressing HSV (oHSV-IL-12), this treatment combination was further explored ([55](#_ENREF_55), [56](#_ENREF_56)). Upon triple therapy with intratumoral oHSV-IL-12 combined with PDCD1 and CTLA4 blockade, profound changes in effector T cells (Teff)/Treg ratios and TAM polarization towards an M1 phenotype were observed. On a functional level, CD4^+^ and CD8^+^ T cells, as well as TAMs, were crucial for tumor rejection. Constitutive intratumoral expression of IL-12 via oHSV is currently being tested in a clinical phase I clinical trial (NCT02062827). To further control local IL-12 production, an adenoviral expression system, which is administered intratumorally and controlled by an oral activator has also been evaluated (Ad-RTS-IL-12) ([57](#_ENREF_57)). A phase I study testing this system in recurrent GBM has recently been completed ([58](#_ENREF_58)). The authors reported encouraging trends regarding overall survival and increased numbers of CD8^+^ cytotoxic T cells in rebiopsied patients. Due to immediate leakage from the expression site, IL-12 triggered systemic IFNG levels which have limited the activator drug dose for a subsequent phase II study (NCT04006119). This study not only demonstrated the potential of local IL-12 therapy in GBM, but also highlighted the necessity of tight spatial and temporal control over IL-12. Local administration via convection-enhanced delivery (CED), a neurosurgical method that allows controlled perfusion of large brain areas with therapeutic agents at defined neuroanatomical locations could be an alternative to gene therapy ([59](#_ENREF_59)). CED is currently employed in preclinical studies to evaluate intratumoral cytokine delivery, replacing osmotic minipumps ([60](#_ENREF_60), [61](#_ENREF_61)). CED has also been successfully applied for antibody delivery directed against the immune checkpoint CD276 or B7H3 in pediatric brain stem tumors ([62](#_ENREF_62)). Modulation of TAM activity via CED-mediated cytokine delivery could further boost current T cell-centric approaches for the treatment of GBM in the future.

***Adoptive cell therapy***

Adoptive cell therapy consists of administering cell products as therapy to patients. Several such approaches, including adoptive NK cells, LAK cells, and TILs, have been tested against solid tumors with modest efficacies. With the exception of TILs, most of these cell therapies have failed to induce regression or show significant clinical benefit in different tumor types ([63-65](#_ENREF_63)). Notwithstanding, isolation and expansion of autologous immune cells from the CNS, especially in the context of GBM, remains a technical challenge ([66](#_ENREF_66)). Far more promising strategies have relied on autologous antigen-specific or engineered T cell therapies whereby peripheral blood mononuclear cells are isolated, expanded, and reinfused to the patient following exposure to specific antigens *in vitro* or engineered to express synthetic receptors, respectively. One such strategy is the use of autologous cytomegalovirus (CMV)-specific T cells, based on the detection of tumor-specific CMV expression in different tumor types and in the majority of GBM tumors ([67](#_ENREF_67), [68](#_ENREF_68)). GBM-targeting CMV-specific T cells are currently under clinical investigation (Supplementary Table 1). However, recent advances in T cell engineering have brought novel strategies to the forefront, such as TCR replacement or chimeric antigen receptor (CAR) T cells directed against specific TAAs ([69](#_ENREF_69)). TCR replacement allows targeting of any cellular protein in an MHC class I-dependent manner. T cells transduced with a TCR specific for H3.3K27M, a mutated form of histone H3, were cytotoxic to H3.3K27M^+^ glioma cells *in vitro* and suppressed tumor progression in intracranial glioma mouse xenografts ([70](#_ENREF_70)). Although currently under clinical investigation, the effectiveness of such TCR replacement approaches in glioma patients requires HLA class I expression. In fact, MHC class I downregulation is an established mechanism of tumor immune evasion ([71](#_ENREF_71)). CAR T cells, on the other hand, are MHC-unrestricted, but limited to targeting surface-expressed molecules. Following the recent success of CAR T cell therapy in the treatment of B cell malignancies, CAR T cells have gained interest as potential immunotherapeutic agents against non-hematological malignancies ([72](#_ENREF_72), [73](#_ENREF_73)). CAR T cell trials in glioma and GBM have recently expanded their targets to a dozen antigens, with epidermal growth factor receptor variant III (EGFRvIII), erb-b2 receptor tyrosine kinase 2 (ERBB2) or HER2, disialoganglioside (GD2) and interleukin 13 receptor subunit alpha 2 (IL13RA2) as the most commonly used ([74-78](#_ENREF_74)) (Supplementary Table 1). These studies have established CAR T cells as safe and potentially efficacious in targeting brain tumors. However, broad clinical application of CAR-based cell therapies for the treatment of solid tumors still faces some limitations, particularly in targeting heterogeneous tumors like GBM, known to harbor cell subpopulations expressing diverse antigen profiles ([79](#_ENREF_79), [80](#_ENREF_80)). Additionally, CAR T cell-mediated selective pressure may drive the outgrowth of antigen-negative clones – a phenomenon termed antigen loss – leading to tumor recurrence, as described in EGFRvIII and IL13RA2 CAR T cell trials([74](#_ENREF_74), [81](#_ENREF_81)). In an attempt to counter this selective advantage, some groups have developed novel CAR T cell designs for the simultaneous targeting of multiple antigens, e.g. tandem CARs and bispecific T cell engagers (BiTEs), or with the release of soluble factors into the TME, e.g. armored CARs and T cells redirected for antigen-unrestricted cytokine-initiated killing (TRUCKs), inducing epitope spreading ([73](#_ENREF_73), [82-84](#_ENREF_82)). Yet, promising approaches like these may carry an increased risk of potential side effects in the form of cytokine release syndrome and unintended reactivity against healthy tissues([85](#_ENREF_85)). Another major obstacle to successful CAR T cell therapy in GBM is maintaining T cell activity in a highly immunosuppressive TME ([86](#_ENREF_86)). Some reports have inclusively described a compensatory immunosuppressive response in the brain characterized by the influx of immunosuppressive factors and Tregs following CAR T cell administration ([74](#_ENREF_74), [87](#_ENREF_87), [88](#_ENREF_88)). Thus, successful CAR T cell therapies in the brain will most likely require engineering of CAR T cells intrinsically resistant to immunosuppression. As new concepts and designs continue to emerge, successful CAR-based therapies for brain tumors will most likely spread to other immune cell types, notably NK cells, MΦ, and MG ([89-91](#_ENREF_89)).

**Supplementary references**

1. Omuro A, Vlahovic G, Lim M, Sahebjam S, Baehring J, Cloughesy T, et al. Nivolumab with or without ipilimumab in patients with recurrent glioblastoma: results from exploratory phase I cohorts of CheckMate 143. *Neuro Oncol* (2018) 20(5):674-86. doi: 10.1093/neuonc/nox208. PubMed PMID: 29106665; PubMed Central PMCID: PMCPMC5892140.

2. Cloughesy TF, Mochizuki AY, Orpilla JR, Hugo W, Lee AH, Davidson TB, et al. Neoadjuvant anti-PD-1 immunotherapy promotes a survival benefit with intratumoral and systemic immune responses in recurrent glioblastoma. *Nat Med* (2019) 25(3):477-86. Epub 2019/02/12. doi: 10.1038/s41591-018-0337-7. PubMed PMID: 30742122; PubMed Central PMCID: PMCPMC6408961.

3. Schalper KA, Rodriguez-Ruiz ME, Diez-Valle R, Lopez-Janeiro A, Porciuncula A, Idoate MA, et al. Neoadjuvant nivolumab modifies the tumor immune microenvironment in resectable glioblastoma. *Nat Med* (2019) 25(3):470-6. Epub 2019/02/12. doi: 10.1038/s41591-018-0339-5. PubMed PMID: 30742120.

4. Zhao J, Chen AX, Gartrell RD, Silverman AM, Aparicio L, Chu T, et al. Immune and genomic correlates of response to anti-PD-1 immunotherapy in glioblastoma. *Nat Med* (2019) 25(3):462-9. Epub 2019/02/12. doi: 10.1038/s41591-019-0349-y. PubMed PMID: 30742119; PubMed Central PMCID: PMCPMC6810613.

5. Banchereau J, Steinman RM. Dendritic cells and the control of immunity. *Nature* (1998) 392(6673):245-52. doi: 10.1038/32588. PubMed PMID: 9521319.

6. Salcedo M, Bercovici N, Taylor R, Vereecken P, Massicard S, Duriau D, et al. Vaccination of melanoma patients using dendritic cells loaded with an allogeneic tumor cell lysate. *Cancer Immunol Immunother* (2006) 55(7):819-29. doi: 10.1007/s00262-005-0078-6. PubMed PMID: 16187085.

7. Jahnisch H, Fussel S, Kiessling A, Wehner R, Zastrow S, Bachmann M, et al. Dendritic cell-based immunotherapy for prostate cancer. *Clin Dev Immunol* (2010) 2010:517493. doi: 10.1155/2010/517493. PubMed PMID: 21076523; PubMed Central PMCID: PMC2975068.

8. Steele JC, Rao A, Marsden JR, Armstrong CJ, Berhane S, Billingham LJ, et al. Phase I/II trial of a dendritic cell vaccine transfected with DNA encoding melan A and gp100 for patients with metastatic melanoma. *Gene Ther* (2011) 18(6):584-93. doi: 10.1038/gt.2011.1. PubMed PMID: 21307889.

9. Rosenblatt J, Vasir B, Uhl L, Blotta S, Macnamara C, Somaiya P, et al. Vaccination with dendritic cell/tumor fusion cells results in cellular and humoral antitumor immune responses in patients with multiple myeloma. *Blood* (2011) 117(2):393-402. doi: 10.1182/blood-2010-04-277137. PubMed PMID: 21030562; PubMed Central PMCID: PMC3031474.

10. de Vries CR, Kaufman HL, Lattime EC. Oncolytic viruses: focusing on the tumor microenvironment. *Cancer Gene Ther* (2015) 22(4):169-71. Epub 2015/02/28. doi: 10.1038/cgt.2015.11. PubMed PMID: 25721204.

11. Martuza RL, Malick A, Markert JM, Ruffner KL, Coen DM. Experimental therapy of human glioma by means of a genetically engineered virus mutant. *Science* (1991) 252(5007):854-6. doi: 10.1126/science.1851332. PubMed PMID: 1851332.

12. Hulou MM, Cho CF, Chiocca EA, Bjerkvig R. Experimental therapies: gene therapies and oncolytic viruses. *Handb Clin Neurol* (2016) 134:183-97. Epub 2016/03/08. doi: 10.1016/B978-0-12-802997-8.00011-6. PubMed PMID: 26948355.

13. Chiocca EA, Abbed KM, Tatter S, Louis DN, Hochberg FH, Barker F, et al. A phase I open-label, dose-escalation, multi-institutional trial of injection with an E1B-Attenuated adenovirus, ONYX-015, into the peritumoral region of recurrent malignant gliomas, in the adjuvant setting. *Mol Ther* (2004) 10(5):958-66. Epub 2004/10/29. doi: 10.1016/j.ymthe.2004.07.021. PubMed PMID: 15509513.

14. Takeuchi O, Akira S. Pattern recognition receptors and inflammation. *Cell* (2010) 140(6):805-20. Epub 2010/03/23. doi: 10.1016/j.cell.2010.01.022. PubMed PMID: 20303872.

15. Shekarian T, Valsesia-Wittmann S, Brody J, Michallet MC, Depil S, Caux C, et al. Pattern recognition receptors: immune targets to enhance cancer immunotherapy. *Annals of Oncology* (2017) 28(8):1756-66. doi: 10.1093/annonc/mdx179.

16. Bowles AP, Jr., Perkins E. Long-term remission of malignant brain tumors after intracranial infection: a report of four cases. *Neurosurgery* (1999) 44(3):636-42; discussion 42-3. Epub 1999/03/09. doi: 10.1097/00006123-199903000-00110. PubMed PMID: 10069601.

17. De Bonis P, Albanese A, Lofrese G, de Waure C, Mangiola A, Pettorini BL, et al. Postoperative infection may influence survival in patients with glioblastoma: simply a myth? *Neurosurgery* (2011) 69(4):864-8; discussion 8-9. Epub 2011/09/09. doi: 10.1227/NEU.0b013e318222adfa. PubMed PMID: 21900810.

18. Chicoine MR, Won EK, Zahner MC. Intratumoral injection of lipopolysaccharide causes regression of subcutaneously implanted mouse glioblastoma multiforme. *Neurosurgery* (2001) 48(3):607-14; discussion 14-5. Epub 2001/03/29. doi: 10.1097/00006123-200103000-00032. PubMed PMID: 11270552.

19. Chicoine MR, Zahner M, Won EK, Kalra RR, Kitamura T, Perry A, et al. The in vivo antitumoral effects of lipopolysaccharide against glioblastoma multiforme are mediated in part by Toll-like receptor 4. *Neurosurgery* (2007) 60(2):372-80; discussion 81. Epub 2007/02/10. doi: 10.1227/01.Neu.0000249280.61761.2e. PubMed PMID: 17290189.

20. Sarrazy V, Vedrenne N, Billet F, Bordeau N, Lepreux S, Vital A, et al. TLR4 signal transduction pathways neutralize the effect of Fas signals on glioblastoma cell proliferation and migration. *Cancer Lett* (2011) 311(2):195-202. Epub 2011/08/20. doi: 10.1016/j.canlet.2011.07.018. PubMed PMID: 21852034.

21. Han S, Wang C, Qin X, Xia J, Wu A. LPS alters the immuno-phenotype of glioma and glioma stem-like cells and induces in vivo antitumor immunity via TLR4. *J Exp Clin Cancer Res* (2017) 36(1):83. Epub 2017/06/24. doi: 10.1186/s13046-017-0552-y. PubMed PMID: 28641579; PubMed Central PMCID: PMCPMC5480420.

22. Grauer OM, Molling JW, Bennink E, Toonen LW, Sutmuller RP, Nierkens S, et al. TLR ligands in the local treatment of established intracerebral murine gliomas. *J Immunol* (2008) 181(10):6720-9. Epub 2008/11/05. doi: 10.4049/jimmunol.181.10.6720. PubMed PMID: 18981089.

23. Ursu R, Carpentier A, Metellus P, Lubrano V, Laigle-Donadey F, Capelle L, et al. Intracerebral injection of CpG oligonucleotide for patients with de novo glioblastoma-A phase II multicentric, randomised study. *Eur J Cancer* (2017) 73:30-7. Epub 2017/02/01. doi: 10.1016/j.ejca.2016.12.003. PubMed PMID: 28142059.

24. El Andaloussi A, Sonabend AM, Han Y, Lesniak MS. Stimulation of TLR9 with CpG ODN enhances apoptosis of glioma and prolongs the survival of mice with experimental brain tumors. *Glia* (2006) 54(6):526-35. Epub 2006/08/15. doi: 10.1002/glia.20401. PubMed PMID: 16906541.

25. Huang B, Zhao J, Unkeless JC, Feng ZH, Xiong H. TLR signaling by tumor and immune cells: a double-edged sword. *Oncogene* (2008) 27(2):218-24. Epub 2008/01/08. doi: 10.1038/sj.onc.1210904. PubMed PMID: 18176603.

26. Alvarado AG, Thiagarajan PS, Mulkearns-Hubert EE, Silver DJ, Hale JS, Alban TJ, et al. Glioblastoma Cancer Stem Cells Evade Innate Immune Suppression of Self-Renewal through Reduced TLR4 Expression. *Cell Stem Cell* (2017) 20(4):450-61 e4. Epub 2017/01/17. doi: 10.1016/j.stem.2016.12.001. PubMed PMID: 28089910; PubMed Central PMCID: PMCPMC5822422.

27. Li C, Ma L, Liu Y, Li Z, Wang Q, Chen Z, et al. TLR2 promotes development and progression of human glioma via enhancing autophagy. *Gene* (2019) 700:52-9. Epub 2019/03/23. doi: 10.1016/j.gene.2019.02.084. PubMed PMID: 30898699.

28. Leng L, Jiang T, Zhang Y. TLR9 expression is associated with prognosis in patients with glioblastoma multiforme. *J Clin Neurosci* (2012) 19(1):75-80. Epub 2011/12/16. doi: 10.1016/j.jocn.2011.03.037. PubMed PMID: 22169598.

29. Mu L, Wang Y, Wang Y, Zhang H, Shang D, Tan F, et al. Tumor Location and Survival Outcomes in Adult Patients with Supratentorial Glioblastoma by Levels of Toll-Like Receptor 9 Expression. *World Neurosurg* (2017) 97:279-83. Epub 2016/10/17. doi: 10.1016/j.wneu.2016.10.015. PubMed PMID: 27744078.

30. Qian J, Luo F, Yang J, Liu J, Liu R, Wang L, et al. TLR2 Promotes Glioma Immune Evasion by Downregulating MHC Class II Molecules in Microglia. *Cancer Immunol Res* (2018) 6(10):1220-33. Epub 2018/08/23. doi: 10.1158/2326-6066.CIR-18-0020. PubMed PMID: 30131377.

31. Dzaye O, Hu F, Derkow K, Haage V, Euskirchen P, Harms C, et al. Glioma Stem Cells but Not Bulk Glioma Cells Upregulate IL-6 Secretion in Microglia/Brain Macrophages via Toll-like Receptor 4 Signaling. *J Neuropathol Exp Neurol* (2016) 75(5):429-40. Epub 2016/04/01. doi: 10.1093/jnen/nlw016. PubMed PMID: 27030742; PubMed Central PMCID: PMCPMC5009477.

32. Wang H, Lathia JD, Wu Q, Wang J, Li Z, Heddleston JM, et al. Targeting interleukin 6 signaling suppresses glioma stem cell survival and tumor growth. *Stem Cells* (2009) 27(10):2393-404. Epub 2009/08/07. doi: 10.1002/stem.188. PubMed PMID: 19658188; PubMed Central PMCID: PMCPMC2825688.

33. Vinnakota K, Hu F, Ku MC, Georgieva PB, Szulzewsky F, Pohlmann A, et al. Toll-like receptor 2 mediates microglia/brain macrophage MT1-MMP expression and glioma expansion. *Neuro Oncol* (2013) 15(11):1457-68. Epub 2013/09/10. doi: 10.1093/neuonc/not115. PubMed PMID: 24014382; PubMed Central PMCID: PMCPMC3813418.

34. Hu F, Ku MC, Markovic D, Dzaye O, Lehnardt S, Synowitz M, et al. Glioma-associated microglial MMP9 expression is upregulated by TLR2 signaling and sensitive to minocycline. *Int J Cancer* (2014) 135(11):2569-78. Epub 2014/04/23. doi: 10.1002/ijc.28908. PubMed PMID: 24752463; PubMed Central PMCID: PMCPMC4519695.

35. Triller P, Bachorz J, Synowitz M, Kettenmann H, Markovic D. O-Vanillin Attenuates the TLR2 Mediated Tumor-Promoting Phenotype of Microglia. *Int J Mol Sci* (2020) 21(8). Epub 2020/04/26. doi: 10.3390/ijms21082959. PubMed PMID: 32331440.

36. Garzon-Muvdi T, Theodros D, Luksik AS, Maxwell R, Kim E, Jackson CM, et al. Dendritic cell activation enhances anti-PD-1 mediated immunotherapy against glioblastoma. *Oncotarget* (2018) 9(29):20681-97. Epub 2018/05/15. doi: 10.18632/oncotarget.25061. PubMed PMID: 29755681; PubMed Central PMCID: PMCPMC5945499.

37. De Waele J, Marcq E, Van Audenaerde JR, Van Loenhout J, Deben C, Zwaenepoel K, et al. Poly(I:C) primes primary human glioblastoma cells for an immune response invigorated by PD-L1 blockade. *Oncoimmunology* (2018) 7(3):e1407899. Epub 2018/02/06. doi: 10.1080/2162402X.2017.1407899. PubMed PMID: 29399410; PubMed Central PMCID: PMCPMC5790389.

38. Deng S, Zhu S, Qiao Y, Liu YJ, Chen W, Zhao G, et al. Recent advances in the role of toll-like receptors and TLR agonists in immunotherapy for human glioma. *Protein Cell* (2014) 5(12):899-911. Epub 2014/11/21. doi: 10.1007/s13238-014-0112-6. PubMed PMID: 25411122; PubMed Central PMCID: PMCPMC4259890.

39. McCarthy EF. The toxins of William B. Coley and the treatment of bone and soft-tissue sarcomas. *The Iowa orthopaedic journal* (2006) 26:154-8. Epub 2006/06/23. PubMed PMID: 16789469; PubMed Central PMCID: PMCPMC1888599.

40. Coley WB. II. Contribution to the Knowledge of Sarcoma. *Annals of surgery* (1891) 14(3):199-220. Epub 1891/09/01. doi: 10.1097/00000658-189112000-00015. PubMed PMID: 17859590; PubMed Central PMCID: PMCPMC1428624.

41. Ganai S, Arenas RB, Sauer JP, Bentley B, Forbes NS. In tumors Salmonella migrate away from vasculature toward the transition zone and induce apoptosis. *Cancer Gene Ther* (2011) 18(7):457-66. Epub 2011/03/26. doi: 10.1038/cgt.2011.10. PubMed PMID: 21436868; PubMed Central PMCID: PMCPMC3117926.

42. Uchugonova A, Zhang Y, Salz R, Liu F, Suetsugu A, Zhang L, et al. Imaging the Different Mechanisms of Prostate Cancer Cell-killing by Tumor-targeting Salmonella typhimurium A1-R. *Anticancer research* (2015) 35(10):5225-9. Epub 2015/09/27. PubMed PMID: 26408681.

43. Mehta N, Lyon JG, Patil K, Mokarram N, Kim C, Bellamkonda RV. Bacterial Carriers for Glioblastoma Therapy. *Mol Ther Oncolytics* (2017) 4:1-17. Epub 2017/03/28. doi: 10.1016/j.omto.2016.12.003. PubMed PMID: 28345020; PubMed Central PMCID: PMCPMC5363759.

44. Mansour M, Ismail S, Abou-Aisha K. Bacterial delivery of the anti-tumor azurin-like protein Laz to glioblastoma cells. *AMB Express* (2020) 10(1):59. Epub 2020/03/30. doi: 10.1186/s13568-020-00995-8. PubMed PMID: 32221741; PubMed Central PMCID: PMCPMC7099546.

45. Chowdhury S, Castro S, Coker C, Hinchliffe TE, Arpaia N, Danino T. Programmable bacteria induce durable tumor regression and systemic antitumor immunity. *Nat Med* (2019) 25(7):1057-63. Epub 2019/07/05. doi: 10.1038/s41591-019-0498-z. PubMed PMID: 31270504; PubMed Central PMCID: PMCPMC6688650.

46. Frahm M, Felgner S, Kocijancic D, Rohde M, Hensel M, Curtiss R, 3rd, et al. Efficiency of conditionally attenuated Salmonella enterica serovar Typhimurium in bacterium-mediated tumor therapy. *mBio* (2015) 6(2). Epub 2015/04/16. doi: 10.1128/mBio.00254-15. PubMed PMID: 25873375; PubMed Central PMCID: PMCPMC4453544.

47. Coers J, Starnbach MN, Howard JC. Modeling infectious disease in mice: co-adaptation and the role of host-specific IFNgamma responses. *PLoS pathogens* (2009) 5(5):e1000333. Epub 2009/05/30. doi: 10.1371/journal.ppat.1000333. PubMed PMID: 19478881; PubMed Central PMCID: PMCPMC2682201.

48. Jain KK. A Critical Overview of Targeted Therapies for Glioblastoma. *Front Oncol* (2018) 8:419. Epub 2018/10/31. doi: 10.3389/fonc.2018.00419. PubMed PMID: 30374421; PubMed Central PMCID: PMCPMC6196260.

49. Zanganeh S, Georgala P, Corbo C, Arabi L, Ho JQ, Javdani N, et al. Immunoengineering in glioblastoma imaging and therapy. *Wiley Interdiscip Rev Nanomed Nanobiotechnol* (2019) 11(6):e1575. Epub 2019/08/14. doi: 10.1002/wnan.1575. PubMed PMID: 31407522.

50. Wang X, Xiong Z, Liu Z, Huang X, Jiang X. Angiopep-2/IP10-EGFRvIIIscFv modified nanoparticles and CTL synergistically inhibit malignant glioblastoma. *Sci Rep* (2018) 8(1):12827. Epub 2018/08/29. doi: 10.1038/s41598-018-30072-x. PubMed PMID: 30150691; PubMed Central PMCID: PMCPMC6110710.

51. Li TF, Xu YH, Li K, Wang C, Liu X, Yue Y, et al. Doxorubicin-polyglycerol-nanodiamond composites stimulate glioblastoma cell immunogenicity through activation of autophagy. *Acta Biomater* (2019) 86:381-94. Epub 2019/01/18. doi: 10.1016/j.actbio.2019.01.020. PubMed PMID: 30654213.

52. Sun T, Patil R, Galstyan A, Klymyshyn D, Ding H, Chesnokova A, et al. Blockade of a Laminin-411-Notch Axis with CRISPR/Cas9 or a Nanobioconjugate Inhibits Glioblastoma Growth through Tumor-Microenvironment Cross-talk. *Cancer Res* (2019) 79(6):1239-51. Epub 2019/01/20. doi: 10.1158/0008-5472.CAN-18-2725. PubMed PMID: 30659021; PubMed Central PMCID: PMCPMC6625517.

53. Zanganeh S, Hutter G, Spitler R, Lenkov O, Mahmoudi M, Shaw A, et al. Iron oxide nanoparticles inhibit tumour growth by inducing pro-inflammatory macrophage polarization in tumour tissues. *Nat Nanotechnol* (2016) 11(11):986-94. Epub 2016/11/01. doi: 10.1038/nnano.2016.168. PubMed PMID: 27668795; PubMed Central PMCID: PMCPMC5198777.

54. Tugues S, Burkhard SH, Ohs I, Vrohlings M, Nussbaum K, Vom Berg J, et al. New insights into IL-12-mediated tumor suppression. *Cell Death Differ* (2015) 22(2):237-46. Epub 2014/09/06. doi: 10.1038/cdd.2014.134. PubMed PMID: 25190142; PubMed Central PMCID: PMCPMC4291488.

55. Vom Berg J, Vrohlings M, Haller S, Haimovici A, Kulig P, Sledzinska A, et al. Intratumoral IL-12 combined with CTLA-4 blockade elicits T cell-mediated glioma rejection. *J Exp Med* (2013) 210(13):2803-11. Epub 2013/11/28. doi: 10.1084/jem.20130678. PubMed PMID: 24277150; PubMed Central PMCID: PMCPMC3865478.

56. Saha D, Martuza RL, Rabkin SD. Macrophage Polarization Contributes to Glioblastoma Eradication by Combination Immunovirotherapy and Immune Checkpoint Blockade. *Cancer Cell* (2017) 32(2):253-67 e5. Epub 2017/08/16. doi: 10.1016/j.ccell.2017.07.006. PubMed PMID: 28810147; PubMed Central PMCID: PMCPMC5568814.

57. Barrett JA, Cai H, Miao J, Khare PD, Gonzalez P, Dalsing-Hernandez J, et al. Regulated intratumoral expression of IL-12 using a RheoSwitch Therapeutic System((R)) (RTS((R))) gene switch as gene therapy for the treatment of glioma. *Cancer Gene Ther* (2018) 25(5-6):106-16. Epub 2018/05/15. doi: 10.1038/s41417-018-0019-0. PubMed PMID: 29755109; PubMed Central PMCID: PMCPMC6021367.

58. Chiocca EA, Yu JS, Lukas RV, Solomon IH, Ligon KL, Nakashima H, et al. Regulatable interleukin-12 gene therapy in patients with recurrent high-grade glioma: Results of a phase 1 trial. *Sci Transl Med* (2019) 11(505). Epub 2019/08/16. doi: 10.1126/scitranslmed.aaw5680. PubMed PMID: 31413142.

59. Barua NU, Gill SS, Love S. Convection-enhanced drug delivery to the brain: therapeutic potential and neuropathological considerations. *Brain Pathol* (2014) 24(2):117-27. Epub 2013/08/16. doi: 10.1111/bpa.12082. PubMed PMID: 23944716.

60. Beffinger M, Schellhammer L, Pantelyushin S, Vom Berg J. Delivery of Antibodies into the Murine Brain via Convection-enhanced Delivery. *J Vis Exp* (2019) (149). Epub 2019/08/06. doi: 10.3791/59675. PubMed PMID: 31380848.

61. Rechberger JS, Power EA, Lu VM, Zhang L, Sarkaria JN, Daniels DJ. Evaluating infusate parameters for direct drug delivery to the brainstem: a comparative study of convection-enhanced delivery versus osmotic pump delivery. *Neurosurg Focus* (2020) 48(1):E2. Epub 2020/01/03. doi: 10.3171/2019.10.FOCUS19703. PubMed PMID: 31896090.

62. Souweidane MM, Kramer K, Pandit-Taskar N, Zhou Z, Haque S, Zanzonico P, et al. Convection-enhanced delivery for diffuse intrinsic pontine glioma: a single-centre, dose-escalation, phase 1 trial. *Lancet Oncol* (2018) 19(8):1040-50. Epub 2018/06/20. doi: 10.1016/S1470-2045(18)30322-X. PubMed PMID: 29914796; PubMed Central PMCID: PMCPMC6692905.

63. Law TM, Motzer RJ, Mazumdar M, Sell KW, Walther PJ, O'Connell M, et al. Phase III randomized trial of interleukin-2 with or without lymphokine-activated killer cells in the treatment of patients with advanced renal cell carcinoma. *Cancer* (1995) 76(5):824-32. Epub 1995/09/01. doi: 10.1002/1097-0142(19950901)76:5<824::aid-cncr2820760517>3.0.co;2-n. PubMed PMID: 8625186.

64. Rosenberg SA, Dudley ME. Cancer regression in patients with metastatic melanoma after the transfer of autologous antitumor lymphocytes. *Proc Natl Acad Sci U S A* (2004) 101 Suppl 2:14639-45. Epub 2004/09/24. doi: 10.1073/pnas.0405730101. PubMed PMID: 15381769; PubMed Central PMCID: PMCPMC521998.

65. Parkhurst MR, Riley JP, Dudley ME, Rosenberg SA. Adoptive transfer of autologous natural killer cells leads to high levels of circulating natural killer cells but does not mediate tumor regression. *Clin Cancer Res* (2011) 17(19):6287-97. Epub 2011/08/17. doi: 10.1158/1078-0432.CCR-11-1347. PubMed PMID: 21844012; PubMed Central PMCID: PMCPMC3186830.

66. Balch CM, Riley LB, Bae YJ, Salmeron MA, Platsoucas CD, von Eschenbach A, et al. Patterns of human tumor-infiltrating lymphocytes in 120 human cancers. *Arch Surg* (1990) 125(2):200-5. Epub 1990/02/01. doi: 10.1001/archsurg.1990.01410140078012. PubMed PMID: 1689143.

67. Cobbs CS, Harkins L, Samanta M, Gillespie GY, Bharara S, King PH, et al. Human cytomegalovirus infection and expression in human malignant glioma. *Cancer Res* (2002) 62(12):3347-50. Epub 2002/06/18. PubMed PMID: 12067971.

68. Schuessler A, Smith C, Beagley L, Boyle GM, Rehan S, Matthews K, et al. Autologous T-cell therapy for cytomegalovirus as a consolidative treatment for recurrent glioblastoma. *Cancer Res* (2014) 74(13):3466-76. Epub 2014/05/06. doi: 10.1158/0008-5472.CAN-14-0296. PubMed PMID: 24795429.

69. Rosenberg SA, Restifo NP, Yang JC, Morgan RA, Dudley ME. Adoptive cell transfer: a clinical path to effective cancer immunotherapy. *Nat Rev Cancer* (2008) 8(4):299-308. Epub 2008/03/21. doi: 10.1038/nrc2355. PubMed PMID: 18354418; PubMed Central PMCID: PMCPMC2553205.

70. Chheda ZS, Kohanbash G, Okada K, Jahan N, Sidney J, Pecoraro M, et al. Novel and shared neoantigen derived from histone 3 variant H3.3K27M mutation for glioma T cell therapy. *J Exp Med* (2018) 215(1):141-57. Epub 2017/12/06. doi: 10.1084/jem.20171046. PubMed PMID: 29203539; PubMed Central PMCID: PMCPMC5748856.

71. de Charette M, Marabelle A, Houot R. Turning tumour cells into antigen presenting cells: The next step to improve cancer immunotherapy? *Eur J Cancer* (2016) 68:134-47. Epub 2016/10/19. doi: 10.1016/j.ejca.2016.09.010. PubMed PMID: 27755997.

72. Maus MV, Grupp SA, Porter DL, June CH. Antibody-modified T cells: CARs take the front seat for hematologic malignancies. *Blood* (2014) 123(17):2625-35. Epub 2014/03/01. doi: 10.1182/blood-2013-11-492231. PubMed PMID: 24578504; PubMed Central PMCID: PMCPMC3999751.

73. D'Aloia MM, Zizzari IG, Sacchetti B, Pierelli L, Alimandi M. CAR-T cells: the long and winding road to solid tumors. *Cell Death Dis* (2018) 9(3):282. Epub 2018/02/17. doi: 10.1038/s41419-018-0278-6. PubMed PMID: 29449531; PubMed Central PMCID: PMCPMC5833816.

74. O'Rourke DM, Nasrallah MP, Desai A, Melenhorst JJ, Mansfield K, Morrissette JJD, et al. A single dose of peripherally infused EGFRvIII-directed CAR T cells mediates antigen loss and induces adaptive resistance in patients with recurrent glioblastoma. *Sci Transl Med* (2017) 9(399). Epub 2017/07/21. doi: 10.1126/scitranslmed.aaa0984. PubMed PMID: 28724573; PubMed Central PMCID: PMCPMC5762203.

75. Ahmed N, Brawley V, Hegde M, Bielamowicz K, Kalra M, Landi D, et al. HER2-Specific Chimeric Antigen Receptor-Modified Virus-Specific T Cells for Progressive Glioblastoma: A Phase 1 Dose-Escalation Trial. *JAMA Oncol* (2017) 3(8):1094-101. Epub 2017/04/21. doi: 10.1001/jamaoncol.2017.0184. PubMed PMID: 28426845; PubMed Central PMCID: PMCPMC5747970.

76. Golinelli G, Grisendi G, Prapa M, Bestagno M, Spano C, Rossignoli F, et al. Targeting GD2-positive glioblastoma by chimeric antigen receptor empowered mesenchymal progenitors. *Cancer Gene Ther* (2018). Epub 2018/11/23. doi: 10.1038/s41417-018-0062-x. PubMed PMID: 30464207.

77. Brown CE, Badie B, Barish ME, Weng L, Ostberg JR, Chang WC, et al. Bioactivity and Safety of IL13Ralpha2-Redirected Chimeric Antigen Receptor CD8+ T Cells in Patients with Recurrent Glioblastoma. *Clin Cancer Res* (2015) 21(18):4062-72. Epub 2015/06/11. doi: 10.1158/1078-0432.CCR-15-0428. PubMed PMID: 26059190; PubMed Central PMCID: PMCPMC4632968.

78. Townsend MH, Shrestha G, Robison RA, O'Neill KL. The expansion of targetable biomarkers for CAR T cell therapy. *J Exp Clin Cancer Res* (2018) 37(1):163. Epub 2018/07/23. doi: 10.1186/s13046-018-0817-0. PubMed PMID: 30031396; PubMed Central PMCID: PMCPMC6054736.

79. Friedmann-Morvinski D. Glioblastoma heterogeneity and cancer cell plasticity. *Crit Rev Oncog* (2014) 19(5):327-36. Epub 2014/11/19. doi: 10.1615/critrevoncog.2014011777. PubMed PMID: 25404148.

80. Little SE, Popov S, Jury A, Bax DA, Doey L, Al-Sarraj S, et al. Receptor tyrosine kinase genes amplified in glioblastoma exhibit a mutual exclusivity in variable proportions reflective of individual tumor heterogeneity. *Cancer Res* (2012) 72(7):1614-20. Epub 2012/02/09. doi: 10.1158/0008-5472.CAN-11-4069. PubMed PMID: 22311673.

81. Brown CE, Alizadeh D, Starr R, Weng L, Wagner JR, Naranjo A, et al. Regression of Glioblastoma after Chimeric Antigen Receptor T-Cell Therapy. *N Engl J Med* (2016) 375(26):2561-9. Epub 2016/12/29. doi: 10.1056/NEJMoa1610497. PubMed PMID: 28029927; PubMed Central PMCID: PMCPMC5390684.

82. Hegde M, Mukherjee M, Grada Z, Pignata A, Landi D, Navai SA, et al. Tandem CAR T cells targeting HER2 and IL13Ralpha2 mitigate tumor antigen escape. *J Clin Invest* (2016) 126(8):3036-52. Epub 2016/07/19. doi: 10.1172/JCI83416. PubMed PMID: 27427982; PubMed Central PMCID: PMCPMC4966331.

83. Choi BD, Yu X, Castano AP, Bouffard AA, Schmidts A, Larson RC, et al. CAR-T cells secreting BiTEs circumvent antigen escape without detectable toxicity. *Nat Biotechnol* (2019) 37(9):1049-58. Epub 2019/07/25. doi: 10.1038/s41587-019-0192-1. PubMed PMID: 31332324.

84. Tokarew N, Ogonek J, Endres S, von Bergwelt-Baildon M, Kobold S. Teaching an old dog new tricks: next-generation CAR T cells. *Br J Cancer* (2019) 120(1):26-37. Epub 2018/11/11. doi: 10.1038/s41416-018-0325-1. PubMed PMID: 30413825; PubMed Central PMCID: PMCPMC6325111.

85. Bonifant CL, Jackson HJ, Brentjens RJ, Curran KJ. Toxicity and management in CAR T-cell therapy. *Mol Ther Oncolytics* (2016) 3:16011. Epub 2016/09/15. doi: 10.1038/mto.2016.11. PubMed PMID: 27626062; PubMed Central PMCID: PMCPMC5008265.

86. Moon EK, Wang LC, Dolfi DV, Wilson CB, Ranganathan R, Sun J, et al. Multifactorial T-cell hypofunction that is reversible can limit the efficacy of chimeric antigen receptor-transduced human T cells in solid tumors. *Clin Cancer Res* (2014) 20(16):4262-73. Epub 2014/06/13. doi: 10.1158/1078-0432.CCR-13-2627. PubMed PMID: 24919573; PubMed Central PMCID: PMCPMC4134701.

87. John LB, Devaud C, Duong CP, Yong CS, Beavis PA, Haynes NM, et al. Anti-PD-1 antibody therapy potently enhances the eradication of established tumors by gene-modified T cells. *Clin Cancer Res* (2013) 19(20):5636-46. Epub 2013/07/23. doi: 10.1158/1078-0432.CCR-13-0458. PubMed PMID: 23873688.

88. Peng W, Liu C, Xu C, Lou Y, Chen J, Yang Y, et al. PD-1 blockade enhances T-cell migration to tumors by elevating IFN-gamma inducible chemokines. *Cancer Res* (2012) 72(20):5209-18. Epub 2012/08/24. doi: 10.1158/0008-5472.CAN-12-1187. PubMed PMID: 22915761; PubMed Central PMCID: PMCPMC3476734.

89. Heczey A, Liu D, Tian G, Courtney AN, Wei J, Marinova E, et al. Invariant NKT cells with chimeric antigen receptor provide a novel platform for safe and effective cancer immunotherapy. *Blood* (2014) 124(18):2824-33. Epub 2014/07/23. doi: 10.1182/blood-2013-11-541235. PubMed PMID: 25049283; PubMed Central PMCID: PMCPMC4215313.

90. Klichinsky M, Ruella M, Shestova O, Lu XM, Best A, Zeeman M, et al. Human chimeric antigen receptor macrophages for cancer immunotherapy. *Nat Biotechnol* (2020). Epub 2020/05/04. doi: 10.1038/s41587-020-0462-y. PubMed PMID: 32361713.

91. Neumann H. Microglia: a cellular vehicle for CNS gene therapy. *J Clin Invest* (2006) 116(11):2857-60. Epub 2006/11/03. doi: 10.1172/JCI30230. PubMed PMID: 17080190; PubMed Central PMCID: PMCPMC1626126.
